# Using Monte Carlo experiments to select meta‐analytic estimators

**DOI:** 10.1002/jrsm.1467

**Published:** 2020-11-17

**Authors:** Sanghyun Hong, W. Robert Reed

**Affiliations:** ^1^ Department of Economics and Finance University of Canterbury Christchurch New Zealand

**Keywords:** estimator performance, experiments, meta‐analysis, Monte Carlo, publication bias, simulation design

## Abstract

The purpose of this study is to show how Monte Carlo analysis of meta‐analytic estimators can be used to select estimators for specific research situations. Our analysis conducts 1620 individual experiments, where each experiment is defined by a unique combination of sample size, effect size, effect size heterogeneity, publication selection mechanism, and other research characteristics. We compare 11 estimators commonly used in medicine, psychology, and the social sciences. These are evaluated on the basis of bias, mean squared error (MSE), and coverage rates. For our experimental design, we reproduce simulation environments from four recent studies. We demonstrate that relative estimator performance differs across performance measures. Estimator performance is a complex interaction of performance indicator and aspects of the application. An estimator that may be especially good with respect to MSE may perform relatively poorly with respect to coverage rates. We also show that the size of the meta‐analyst's sample and effect heterogeneity are important determinants of relative estimator performance. We use these results to demonstrate how these observable characteristics can guide the meta‐analyst to choose the most appropriate estimator for their research circumstances.

**Highlights**

- Despite much previous research, meta‐analysts do not have much guidance when it comes to selecting a “best” estimator
- This study shows how Monte Carlo experiments can be used to select the “best” estimator for a given research situation
- We compare 11 estimators commonly used in medicine, psychology, and the social sciences
- The estimators are evaluated on four performance measures: bias, mean squared error (MSE), coverage rates, and Type I error rates.
- We conduct 1620 individual experiments, where an experiment is defined by a unique combination of sample size, effect size, effect size heterogeneity, publication selection mechanism, and other research characteristics
- Estimators that are relatively good on one performance measure may perform relatively poorly on another
- The size of the meta‐analyst's sample and effect heterogeneity are important determinants of relative estimator performance
- We demonstrate how the observable characteristics of sample size and effect heterogeneity can guide the meta‐analyst to select the most appropriate estimator for their research circumstances

## 1 INTRODUCTION

Meta‐analysts have an embarrassment of riches when it comes to choosing an estimator for measuring mean effects. The list of potential estimators is long and growing. Accordingly, a literature has arisen that attempts to provide guidance to those seeking a “best” estimator. The purpose of this study is not to produce yet another attempt at recommending estimators. Instead, this study lays out a procedure for how one can identify a best estimator for a given research application. While we provide an example of how such a procedure could work, the purpose of the example is to demonstrate the feasibility and practicality of our approach.

Selecting a best estimator for meta‐analysis (MA) is complicated. “Best” depends on the meta‐analyst's goals. Different meta‐analysts can have different goals. Further, no estimator performs well in every situation. Yet relatively little is known about the circumstances which would cause a given estimator to perform better than others. In an ideal world, there would exist a flow‐chart that would guide researchers toward the estimator that was best for their research application. Given the current state of knowledge, it is not even clear what factors should be included in such a flow‐chart. This study attempts to make progress on this issue. In the remainder of this introduction, we elaborate on what this study does and the results that we obtain.

Our study performs the largest, most extensive Monte Carlo analysis of MA estimators to date. We conduct 1620 individual experiments, where each experiment is defined by a specific combination of sample size (ie, number of estimates in the meta‐analyst's sample), effect size, effect size heterogeneity, publication selection mechanism, and other research characteristics. We compare 11 estimators commonly used in medicine, psychology, and the social sciences. We assess these estimators on the basis of bias, mean squared error (MSE), coverage rates, and Type I error rates.

Our Monte Carlo experiments reproduce experimental designs from four previous studies: Stanley et al^1^; Alinaghi and Reed^2^; Bom and Rachinger^3^; and Carter et al.^4^ We do this rather than design our own experiments for two reasons. Monte Carlo experiments are by definition artificial representations of a complex reality. They involve a large number of subjective judgments. We wanted to select designs that had to some extent been approved by the peer review process. We also wanted to use multiple experimental designs to see if results would differ across simulation environments.

Our research produces four major findings. First, estimators that rank relatively high in terms of average performance on one criterion frequently do not perform as well on other criteria. From this we conclude that meta‐analysts need to prioritize which criteria (Bias, MSE, etc.) are most important to them. Second, estimators that perform relatively well in one experimental design often do not perform as well in others. We identify two possible reasons for this difference across experimental designs, though more research needs to be done to better understand why this is so.

Third, we show that effect size heterogeneity and the number of estimates in the meta‐analyst's sample (“sample size”) are important determinants of estimator performance. Both these characteristics are observable to the meta‐analyst. As such, they can serve as elements of a “flow‐chart” that allows the meta‐analyst to match experimental results to their own research situation, and thus guide them to the best estimator for the problem at hand.

Lastly, we give a specific example of how this would work. Our example assumes that the meta‐analyst wants an estimator that minimizes MSE. The meta‐analyst's sample consists of 100 estimates characterized by a high degree of effect heterogeneity. Further, they believe that the Monte Carlo design of Carter et al^4^ most closely matches their own research situation. The meta‐analyst gathers all the experimental results associated with the Carter et al^4^ experimental design having sample sizes of 100 and a high degree of heterogeneity. They then compare MSE performance across all 11 estimators for this set of experiments and select a “best” estimator, which can then be used for their own research.

Our study proceeds as follows. Section 2 describes the estimators that we study. Section 3 highlights the main characteristics of the different simulation environments used for our analysis. Section 4 defines the performance measures. Section 5 presents our results. Section 6 gives an example of how Monte Carlo experimental results can be used to guide the selection of a “best” estimator for a given research situation. Section 7 concludes with a summary of our main results and suggestions for future research. All of the programming code and output files associated with this project are available at https://osf.io/pr4mb/. We note that our code borrows considerably from Carter et al.^5^

## 2 THE ESTIMATORS

As noted by Carter et al,^4^ ^(p117)^ while many studies have analyzed the performance of meta‐analytic estimators, “…there is very little overlap among these studies in either the methods they have examined or the simulated conditions they have explored.” Table 1 summarizes a selection of previous Monte Carlo studies and compares them in terms of the number of experiments and estimators studied. Our study analyses and compares the performance of 11 estimators. This compares favorably with previous studies both in terms of number of estimators and variety in the types of estimators. We chose our estimators because they either are widely used in the MA literature, or have recently appeared in prominent publications.

**TABLE 1:** Summary of selected Monte Carlo studies of estimator performance: number of experiments and estimators studied

Study	Experiments	Estimators
Stanley et al^1^	180	RE, WLS, WAAP, PP
Alinaghi and Reed^2^	74	WLS‐FE, WLS‐RE, PP
Bom and Rachinger^3^	215	FE, RE, WAAP, PP, EK
Carter et al^4^	432	TF, pC, pU, RE, 3PSM WAAP, PP
Hedges and Vevea^6^	176	5PSM
McShane et al^7^	125	pC, pU, 3PSM
Moreno et al^8^	240	TF(FE‐FE), TF(FE‐RE), TF(RE‐RE), FE, RE, FE‐se, RE‐se, D‐se, FE‐var, RE‐var, D‐var, Harbord, Peters, and Harbord‐C
Reed^9^	36	OLS, PET, PEESE, FE, WLS, RE
Rucker et al^10^	36	TF, CSM, RE, LMA
Simonsohn et al^11^	30	TF, pC, FE
Stanley^12^	120	WLS, FE, PP
Stanley and Doucouliagos^13^	60	FE, RE, Top10, PEESE, PP, WLS‐se, WLS‐Quadratic, WLS‐Cubic
van Aert et al^14^	25	pC, pU, FE, RE
van Assen et al^15^	36	FE, TF, pU, TES
*Our study*	*1620*	*TF, pC, pU, RE, 3PSM, 4PSM, AK1, AK2, WAAP, PP, EK*

*Note:* Estimators: 3PSM/4PSM/5PSM = Three‐Parameter, Four‐Parameter, and Five Parameter Selection Models AK1 = Andrews and Kasy's^16^ “symmetric selection” model AK2 = Andrews and Kasy's^16^ “asymmetric selection” selection CSM = Copas selection model (Copas^25^) EK = Bom and Rachinger's^3^ Endogenous Kink estimator FE = Fixed Effects FE‐se, RE‐se, and WLS‐se/D‐se/PET = Estimates the following model using FE, RE, and WLS: ${\hat{\text{effect}}}_{i}=\alpha +\beta \mathrm{se}\left(\hat{\text{effect}}\right)+{ɛ}_{i}$
FE‐var, RE‐var, and PEESE/D‐var/ = Estimates the following model using FE, RE, and WLS. ${\hat{\text{effect}}}_{i}=\alpha +\beta {\mathrm{se}}^{2}\left(\hat{\text{effect}}\right)+{ɛ}_{i}$
Harbord/Harbord‐C = Harbord, Egger, and Sterne's^26^ “Regression test for small‐study effects” and variant LMA = Limit meta‐analysis (Rucker et al^10^). OLS = OLS regression of estimated effects on a constant. pC = p‐curve pU = p‐uniform Peters = Peters et al's^27^ “Regression test for funnel asymmetry” PP = PET‐PEESE (Stanley and Doucouliagos^22^) RE = Random Effects TES = Test for excess significance (Ioannidis and Trikalinos^28^) TF/TF(RE‐RE) = Trim and Fill with RE used for both the “trim” and “fill” components TF(FE‐FE)/TF(FE‐RE) = Trim and Fill with variants depending on whether FE or RE is used for the “trim” and “fill” components, respectively Top10 = Estimator which uses only the most precise 10% of estimates (Stanley et al.^29^) WLS/WLS‐FE = Weighted Least Squares with weights $\left(\frac{1}{{\mathrm{SE}}_{i}^{2}}\right)$
WLS‐RE = Weighted Least Squares with weights $\left(\frac{1}{{\mathrm{SE}}_{i}^{2}+\tau^{2}}\right)$
WLS‐Quadratic ^=^ Estimates the following model using WLS: ${\hat{\text{effect}}}_{i}=\alpha +\beta_{1}\mathrm{se}\left(\hat{\text{effect}}\right)+\beta_{2}{\mathrm{se}}^{2}\left(\hat{\text{effect}}\right)+{ɛ}_{i}$
WLS‐Cubic = Estimates the following model using WLS: ${\hat{\text{effect}}}_{i}=\alpha +\beta_{1}\mathrm{se}\left(\hat{\text{effect}}\right)+\beta_{2}{\mathrm{se}}^{2}\left(\hat{\text{effect}}\right)+\beta_{3}{\mathrm{se}}^{3}\left(\hat{\text{effect}}\right)+{ɛ}_{i}$
WAAP = Stanley et al's^1^ Weighted Average of the Adequately Powered‐WLS‐FE hybrid estimator.

### 2.1 The context

The estimators are best described within a research context. The following example focuses on a linear regression model, but is easily extended to analyses involving Cohen's *d* and Log‐Odds/logistic regression. Suppose a researcher is interested in synthesizing the results of an empirical literature. The literature consists of studies that estimate the effect of *X* on *Y* using the following linear regression model,

(1) $$Y_{\mathrm{it}}=\alpha_{i}+\beta_{i}X_{\mathrm{it}}+\sum_{k}\gamma_{\mathrm{ikt}}Z_{\mathrm{ikt}}+{ɛ}_{\mathrm{it}},t=1,2,\ldots T_{i},$$

where *i* identifies a given regression having *T*_*i*_ observations. The true effect of *X* on *Y* in any given regression is given by *β*_*i*_. *β*_*i*_ can differ across regressions for many reasons that are unobservable to the meta‐analyst. The distribution of the population effect *β*_*i*_ across regressions is represented by *β*_*i*_~*N*(*μ*, *τ*^2^), *τ*^2^ ≥ 0.

Let ${\hat{\beta }}_{i}$ be the estimated effect from regression *i*. The meta‐analyst collects a sample of estimates, ${\hat{\beta }}_{i}$, *=1,2*,…,*N*, and wants to estimate *μ*, the population mean effect of *X* on *Y*. They know that publication selection may distort their sample of estimates. They have the following estimators available to them: Trim‐and‐Fill, p‐curve, p‐uniform, Random Effects, Three‐Parameter and Four‐Parameter Selection Models, Andrews and Kasy's^16^ “symmetric selection” and “asymmetric selection” models, the Weighted Average of the Adequately Powered‐WLS hybrid estimator, PET‐PEESE, and Bom and Rachinger's^3^ Endogenous Kink estimator. Each of these is briefly described below.

### 2.2 Trim and Fill (TF)

Trim and Fill (Duval and Tweedie^17^) is a method that assumes that any asymmetry in the distribution of effect sizes and SEs is due to publication selection. The method works by iteratively removing individual observations until symmetry in the distribution of effect sizes and SEs is achieved. The removed observations are then added back into the sample, along with artificially generated effect/SE observations that are the mirror images of the removed observations. This ensures that the reconstructed MA sample achieves symmetry. Our estimates are obtained using the *metafor* package in R.

### 2.3 p‐curve (pC)/p‐uniform (pU)

The p‐curve (Simonsohn et al^11^) and p‐uniform (van Assen et al^15^) methods are conceptually identical and similar in implementation. Both estimate the mean true effect from the sample of MA estimates that are statistically significant; that is, have *P*‐values less than 5%. Both assume that estimates with *P*‐values less than .05 are equally likely to be published, and that the respective *P*‐values are independently distributed. Both methods work from the starting point that the distribution of *P*‐values (the “p‐curve”) will be uniformly distributed between 0 and .05 if the null hypothesis is true. Larger, positive effects produce a right skewness to the shape of the “p‐curve.” Neither is recommended in the presence of effect size heterogeneity (van Aert et al^14^).

Conceptually, both methods estimate the value of the true (unobserved) effect that would produce a “p‐curve” closest to the observed “p‐curve.” Both define a loss function that measures the distance between the (transformed) expected and the observed p‐curves and choose a mean true effect that minimizes that loss function. The two methods differ in the metric they use to measure distance. P‐curve uses the Kolmogorov‐Smirnov test statistic as a distance metric, while p‐uniform's metric is based on the Irwin‐Hall distribution. They also differ in that the p‐curve estimator does not produce a SE. We follow standard practice and only include significant estimates that are same‐signed (positive in our case). Our p‐curve estimates are obtained from the programming code in Carter et al.^5^ Our p‐uniform estimates use method one in the *puniform* package in R.

### 2.4 Random effects (RE)

The random effects estimator is arguably the most commonly used meta‐analytic estimator. It does not explicitly correct for publication selection other than giving greater weight to more precise estimates of *β*_*i*_. It estimates the population mean effect *μ* assuming the following specification:

(2) $${\hat{\beta }}_{i}=\mu +\varepsilon_{i},i=1,2,\ldots ,N,$$

where $\varepsilon_{i}∼N\left(0,\sigma_{{\hat{\beta }}_{i}}^{2}+\tau^{2}\right)$, $\sigma_{{\hat{\beta }}_{i}}^{2}$ is the variance in ${\hat{\beta }}_{i}$ due to sampling error, and *τ*^2^ is the variance of true effects across studies. $\sigma_{{\hat{\beta }}_{i}}^{2}$ is estimated by ${\mathrm{SE}}_{i}^{2}$, where *SE*_*i*_ is the (estimated) SE of the estimated effect, ${\hat{\beta }}_{i}$. A variety of procedures have been developed to estimate *τ*^2^. Our RE estimates are obtained using the R package *metafor*, where ${\hat{\tau }}^{2}$ is calculated using the restricted maximum likelihood method.

### 2.5 Three‐parameter and four‐parameter selection models (3PSM and 4PSM)

**FIGURE 1:**
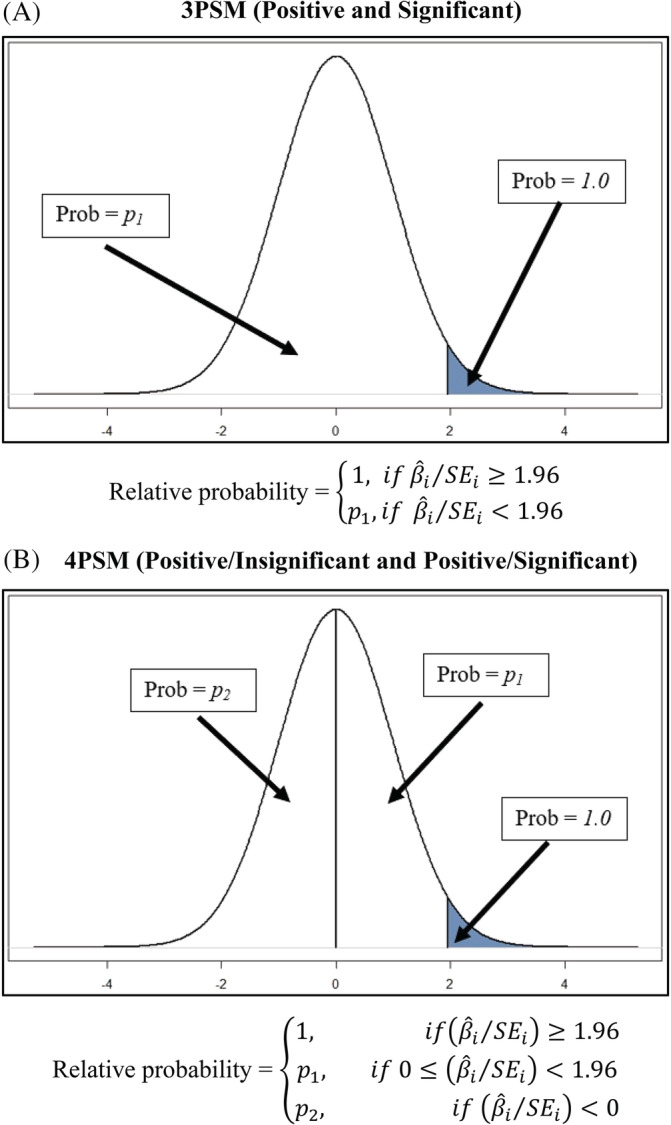
Illustration of 3PSM and 4PSM. [Colour figure can be viewed at wileyonlinelibrary.com]

A variety of selection models have been proposed in the literature to correct for publication bias (Iyengar and Greenhouse^18^; Vevea and Hedges^19^; Vevea and Woods^20^). A common model is the Three‐Parameter Selection Model (3PSM). 3PSM assumes that standardized effect sizes (${\hat{\beta }}_{i}/{\mathrm{SE}}_{i}$) are distributed normally in the population. Publication selection induces differential probabilities of being published, with publication probabilities following a step function. The general method allows researchers to set the values of the steps. For our 3PSM analysis, we follow Carter, Schönbrodt, Gervais, and Hilgard^4^ in allocating estimates to two categories depending on whether the estimates are (a) correctly signed (positive) and statistically significant, $\left({\hat{\beta }}_{i}/{\mathrm{SE}}_{i}\right)\geq 1.96$; or (b) not correctly signed and significant, $\left({\hat{\beta }}_{i}/{\mathrm{SE}}_{i}\right)<1.96$. These have relative publication probabilities equal to 1 and *p*_*1*_, respectively (see Panel A of Figure 1). The “Three‐Parameters” correspond to the mean true effect (*μ*), the extent of effect heterogeneity (*τ*^2^), and *p*_*1*_.

We also consider a Four‐Parameter Selection Model. Our 4PSM adds another category to the 3PSM model: positive and insignificant estimates. The respective categories then become (a) $\left({\hat{\beta }}_{i}/{\mathrm{SE}}_{i}\right)\geq 1.96$; (b) $0\leq \left({\hat{\beta }}_{i}/{\mathrm{SE}}_{i}\right)<1.96$; and (c) $\left({\hat{\beta }}_{i}/{\mathrm{SE}}_{i}\right)<0$.^1^ The associated relative publication probabilities are equal to 1, *p*_*1*_, and *p*_*2*_ (see Panel B of Figure 1); with *μ*, *τ*^2^, *p*_*1*_, and *p*_*2*_ accounting for the Four‐Parameters. We use R's *weightfunct* package to estimate 3PSM and 4PSM. When the relative probabilities of being published are equal to one (ie, no publication selection), these models collapse to the RE model.

### 2.6 AK1 and AK2

**FIGURE 2:**
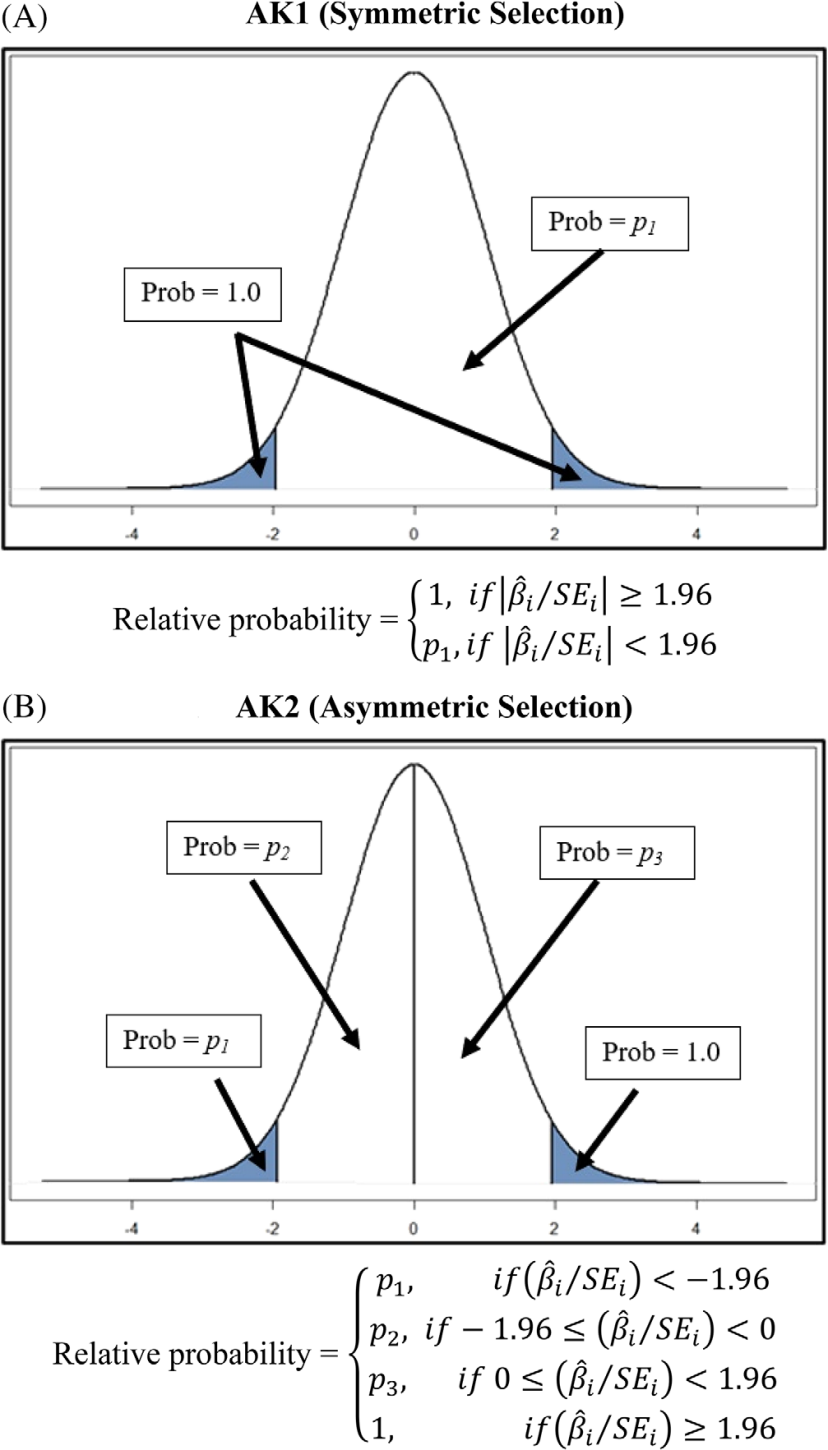
Illustration of AK1 and AK2 [Colour figure can be viewed at wileyonlinelibrary.com]

Similar to 3PSM and 4PSM are two new estimators from Andrews and Kasy.^16^ Like 3PSM and 4PSM, these models categorize estimated effects into groups with different probabilities of being published. The AK1 model groups estimates into significant and insignificant estimates without respect to sign: (i) $\left|{\hat{\beta }}_{i}/{\mathrm{SE}}_{i}\right|\geq 1.96$; and (ii) $\left|{\hat{\beta }}_{i}/{\mathrm{SE}}_{i}\right|<1.96$. Andrews & Kasy refer to this as the “symmetric selection” case (see Panel A of Figure 2). The relative probability that a significant estimate is published is fixed at 1, while estimates that are insignificant are published with probability *p*_*1*_.

Andrews and Kasy^16^ propose another estimator that recognizes that the sign of the estimated effect may also affect selection. The AK2 estimator allocates estimates into four groups: (a) $\left({\hat{\beta }}_{i}/{\mathrm{SE}}_{i}\right)\geq 1.96$, (b) $\left({\hat{\beta }}_{i}/{\mathrm{SE}}_{i}\right)<-1.96$, (c) $-1.96\leq \left({\hat{\beta }}_{i}/{\mathrm{SE}}_{i}\right)<0,$ and (d) $0\leq \left({\hat{\beta }}_{i}/{\mathrm{SE}}_{i}\right)<1.96$. These have relative publication probabilities equal to 1, *p*_*1*_, *p*_*2*_, and *p*_*3*_ (see Panel B of Figure 2). Andrews and Kasy^16^ call this the “asymmetric selection” case. Because the *P*‐values produced by AK1 and AK2 are based on t‐statistics, they require four and six observations, respectively, in order to obtain *P*‐values for all the parameter estimates. This can be a problem for meta‐analyses with very small samples, such as is common in medicine. We use the programming code that accompanies Andrews and Kasy^16^ to obtain our AK1 and AK2 estimates.^2^

### 2.7 Weighted average of the adequately powered‐weighted least squares hybrid estimator (WAAP)

The Weighted Average of the Adequately Powered‐Weighted Least Squares hybrid estimator was introduced in Stanley et al.^1^ Conceptually, this estimator chooses a subset of the *N* estimates ${\hat{\beta }}_{i}$ that are “adequately powered,” defined as coming from regression equations having a power of at least 80%. Weighted Least Squares (weights = $\frac{1}{{\mathrm{SE}}_{i}^{2}}$) is used to estimate Equation (2) in order to obtain an initial estimate of *μ*.

To determine whether a particular estimate comes from an “adequately powered” regression equation, the WAAP estimator determines a threshold value, *δ*, for the effect SE:

(3) $$\delta =\frac{\left|\hat{\mu }\right|}{2.8},$$

where $\hat{\mu }$ is the WLS estimate of *μ* in Equation (2) based on the full sample of *N* estimated effects. Note that this initial estimate of *μ* does not correct for publication bias. WAAP then selects all the ${\hat{\beta }}_{i}\prime s$ for which *SE*_*i*_ < *δ*. Let *M ≤ N* of the ${\hat{\beta }}_{i}\prime s$ satisfy this criterion. It then uses WLS to re‐estimate Equation (2) using only the *M* estimates (the “adequately powered” estimates) to obtain a revised estimate of *μ*. A problem can arise when there too few effect estimates that are adequately powered. Following Stanley et al^1^ if there are fewer than two adequately powered effect estimates, the WAAP estimator uses the WLS estimate from the full sample of *N* estimated effects.

### 2.8 PET‐PEESE (PP)

PET‐PEESE stands for Precision Effect Test and Precision Effect Estimate with SE (Stanley and Doucouliagos^22^). The PP estimator proceeds in two steps. The first step estimates a publication‐corrected variant of Equation (2) using WLS:

(4a) $${\hat{\beta }}_{i}=\mu +\rho ∙{\mathrm{SE}}_{i}+\varepsilon_{i},i=1,2,\ldots ,N.$$

with weights equal to $\frac{1}{{\mathrm{SE}}_{i}^{2}}$. It then tests whether *μ* = 0. If it fails to reject this hypothesis, then PP takes $\hat{\mu }$ as an estimate of *μ*. If it rejects *μ* = 0, it then estimates

(4b) $${\hat{\beta }}_{i}=\mu +\rho ∙{\mathrm{SE}}_{i}^{2}+\varepsilon_{i},i=1,2,\ldots ,N.$$

The estimate of *μ* from Equation (4b) then becomes the updated PP estimate of *μ*.^3^ Following Stanley's^12^ recommendation, we use a one‐tailed test when testing *μ* = 0. While endogeneity lies outside the purview of our study, it should be noted that PET‐PEESE, unlike the other estimators, easily accommodates IV methods.

### 2.9 Endogenous Kink (EK)

Bom and Rachinger^3^ recently proposed a modification to the PET‐PEESE model. The modification concerns a nonlinearity between the size of the bias due to publication selection and the SE. When *μ* is nonzero there is no publication selection when *SE* is very small because all or virtually all estimates are statistically significant. As *SE* increases, the degree of publication selection increases. This induces a non‐linearity in the relationship between bias and SE. This nonlinearity is the reason why Stanley and Doucouliagos^22^ propose including *SE* ^*2*^ in Equation (4b).

As an alternative, Bom and Rachinger^3^ propose the following kinked regression specification:

(5) $${\hat{\beta }}_{i}=\mu +\rho ∙\left[{\mathrm{SE}}_{i}-a\right]I_{\mathrm{SE}\geq a}+\varepsilon_{i},i=1,2,\ldots ,N.$$

where *I*_*SE* ≥ *a*_ is a dummy variable that takes the value 1 whenever *SE* is larger than a cut‐off point *a*. This induces a kink at *SE* = *a*. To determine *a*, Bom and Rachinger^3^ follow a two‐step procedure. They first estimate *μ* as if one was implementing the first stage of the PET‐PEESE procedure.

Assuming the estimated effect is positive, they then calculate the lower bound of a 95% confidence interval around $\hat{\mu }$ where the SE is derived from a RE model (to accommodate effect heterogeneity): $\hat{\mu }-1.96∙\sqrt{{\mathrm{SE}}_{i}^{2}+{\hat{\tau }}^{2}}$. The cutoff value *a* is the value of *SE* that satisfies the equality $\hat{\mu }-1.96∙\sqrt{{\mathrm{SE}}_{i}^{2}+{\hat{\tau }}^{2}}=1.96∙SE_{i}$. Below *a*, most estimates of *μ* are likely to be statistically significant and thus unaffected by publication selection. Beyond *a*, publication selection is likely to become an increasing problem, causing the bias to be linearly related to *SE*. To estimate the EK model, we use programming code provided by Bom and Rachinger.

## 3 THE SIMULATION ENVIRONMENTS

To assess the 11 estimators above, we reproduce the simulation designs from four recently published studies: Stanley et al,^1^ Alinaghi and Reed,^2^ Bom and Rachinger,^3^ and Carter et al.^4^ We chose to work with multiple simulation environments in light of Carter et al^4^ ^(p117)^ assessment of previous research:

Different simulation studies have implemented bias differently, have drawn sample sizes from different distributions, and have varied widely in the value and form of the simulated true underlying effects. This lack of overlap is not surprising given that there is an effectively infinite number of possible combinations of different conditions to explore and no way of determining which conditions actually underlie real‐world data. In other words, not only is there an inherent dimensionality problem in these simulation studies, but there is also no ground truth. These problems are often not discussed in reports of simulation studies, and indeed, many of the reports just cited—explicitly or implicitly—recommended the use of a single method, despite the fact that each study examined performance of only a handful of correction methods in only a limited subset of possible conditions.

Working with multiple simulation environments allows us to determine the sensitivity of our results to alternative experimental designs.

Our choice of simulation environments was made to ensure that we covered scenarios of interest to multiple disciplines. Stanley et al^1^ was published in *Statistics in Medicine*. Carter et al^4^ was published in *Advances in Methods and Practices in Psychological Science*. Alinaghi and Reed^2^ and Bom and Rachinger^3^ were recently published in *Research Synthesis Methods*. Each of the simulation designs are briefly described below. More extensive discussions can be found in the original articles. While the simulation designs cover many different scenarios, many relevant scenarios are missing from the designs.

### 3.1 Stanley, Doucouliagos, and Ioannidis

SD&I^1^ consider two scenarios where researchers are interested in determining the effect of a given treatment, *treat* = {0, 1}. In the “Log Odds Ratio” scenario, primary studies track the effect of a treatment on a binary indicator of “success.” Individual observations are simulated such that the probability of “success” (Y = 1) is 10% for the control group, and (10% + a fixed effect + a mean zero, random component) for the treatment group. Effect heterogeneity is regulated by the variance of the random component, $\sigma_{h}^{2}$.

Primary studies estimate a logistic regression to determine the effect of the treatment on Prob(Y = 1). The parameter of interest is the coefficient on *treat*, *α*_1_. Each study produces a single estimated effect. Variation in the SE of the estimated effects across studies is generated by allowing the primary studies to have different numbers of observations. The mean effect of treatment across all studies, *α*_1_, equals 0.0, 0.30, or 0.54, depending on the experiment. Sample sizes for the simulated meta‐analyses vary across experiments and are pre‐determined to consist of 5, 10, 20, 40, or 80 estimated effects. In the absence of publication selection, a regression of the estimated treatment effects on a constant should produce an unbiased estimate of *α*_1_ in any given MA sample.

Publication selection consists of two regimes: no publication selection, or 50% publication selection. Under 50% publication selection, estimates are sequentially evaluated for inclusion in the meta‐analyst's sample. Each estimate has a 50% chance of being “selected.” If it avoids selection, the estimate is “published” without consideration to its sign and statistical significance. If selected, the estimate is “published” if it is positive and significant. If not, new estimates are generated until a positive and significant estimate is found. This continues until the meta‐analyst's sample attains its pre‐determined size (see Panel A of Appendix 1 in Data S1).^4^

In the second scenario, “Cohen's *d*,” primary studies estimate the effect of a treatment, but this time the dependent variable is continuous. The difference in outcomes between the treatment and control groups is equal to a fixed effect, *α*_1_, plus a random component that differs across studies. Effect heterogeneity is introduced through this random component, which is regulated by the parameter $\sigma_{h}^{2}$.

Each primary study calculates Cohen's *d*, which is the standardized difference in the mean outcome values across the two groups. The mean value of *d* across studies is set equal to either 0 or 0.5, depending on the experiment. Differences in the SEs of *d* are generated by allowing the simulated primary studies to have different sample sizes. In the absence of publication selection, a regression of the estimated treatment effects on a constant will produce an unbiased estimate of the population mean of *d*. Sample sizes for the simulated meta‐analyses are pre‐determined to consist of 5, 10, 20, 40, or 80 estimated effects, depending on the experiment. The Cohen's *d* experiments include the no publication selection and 50% publication selection scenarios used for the Log OR scenario, plus one more: 75/100% publication selection. Under 75/100% publication selection, positive and statistically significant estimates are selected with probability 75%, but 100% of the estimates are restricted to be positive (see Panel B of Appendix 1 in Data S1).

### 3.2 Alinaghi and Reed

A&R^2^ study univariate regression models where a variable *X* affects a continuous variable *Y*. The parameter of interest is the coefficient on *X*. In the “Random Effects” data environment, each study produces one estimate and the population effect differs across studies. The coefficient on *X* equals a fixed component, *α*_1_, plus a random component that is fixed within a study but varies across studies. The overall mean effect of *X* on *Y* is given by *α*_1_. To estimate *α*_1_, the meta‐analyst regresses the study specific estimates on a constant. In the absence of publication selection, the resulting estimate will be unbiased.

A distinctive feature of A&R's experiments is that they fix the size of the sample of estimated effects before publication selection, rather than after. The size of the meta‐analyst's sample is thus determined endogenously, and is affected by the size of the effect. For example, very large population effects will be subject to relatively little publication selection as most estimates will satisfy the selection criteria, whether it be statistical significance or correct sign.

Another distinctive feature of A&R's experiments is that they separate statistical significance from the sign of the estimated effect as criteria for selection. Other studies commonly combine these two, assuming a mechanism that selects estimates that are both positive and statistically significant. A&R's experiments accommodate the fact that these two criteria have different, sometimes conflicting, consequences for estimator performance. All significant/correctly‐signed estimates are “published,” while insignificant/wrong‐signed estimates only have a 10% chance of getting published.

A&R design their simulations to be representative of meta‐analyses in economics and business. These typically have samples of several hundred estimates and substantial effect heterogeneity. In addition to the “Random Effects” data environment described above, A&R also construct a “Panel Random Effects” data environment, where each study has 10 estimates. This models the fact that the overwhelming share of meta‐analyses in economics and business have multiple estimates per study. Effect estimates and SEs are simulated to be more similar within studies than across studies. Publication selection targets the study rather than individual estimates. To be included in the meta‐analyst's sample, a study must have at least 7 out of the 10 estimates be significant/correctly signed.^5^

### 3.3 Bom and Rachinger

B&R^3^ consider univariate regression environments where researchers are interested in estimating the effect of a variable *X*_*1*_ on a dependent variable *Y*, represented by the parameter *α*_1_. Variation in the SEs of estimated effects is accomplished by allowing sample sizes to differ across primary studies. Effect heterogeneity is introduced via an omitted variable (*X*_*2*_) that is correlated with *X*_*1*_. The coefficient on the omitted variable, *α*_2_, is randomly distributed across studies with mean zero and variance $\sigma_{h}^{2}$. Individual estimates of *α*_1_ will be biased for nonzero values of *α*_2_. In the population of all studies, the omitted variable bias averages out. However, publication selection induces a bias in the meta‐analyst's sample when selection depends on the sign and significance of ${\hat{\alpha }}_{1}$.

The experiments are designed to produce 5, 10, 20, 40, or 80 “studies” for a given simulated MA, with each study consisting of one estimated effect. In the absence of publication selection, the regression on a constant produces an unbiased estimate of *α*_1_, where *α*_1_ equals either 0 or 1 depending on the experiment. Publication selection consists of four regimes: no publication selection, 25%, 50%, and 75% publication selection. The publication selection algorithm is modeled after SD&I's 50% publication selection algorithm (see Panel A of Appendix 1 in Data S1).

### 3.4 Carter, Schönbrodt, Gervais, and Hilgard

In the simulation environment of CSG&H^4^ (for Carter, Schönbrodt, Gervais, and Hilgard), primary studies estimate the effect of a treatment using Cohen's *d* as their measure of effect. The difference in outcomes for the treatment and control groups is equal to a fixed effect, *α*_1_, plus a random component that differs across studies. Effect heterogeneity is introduced through this random component, which is regulated by the parameter $\sigma_{h}^{2}$. The mean value of *d* takes on four values depending on the experiment: 0, 0.2, 0.5, and 0.8. Differences in the SEs of *d* for a given experiment are generated by allowing the simulated primary studies to have different sample sizes.

CSG&H introduce two types of distortions in the research environment. They employ a publication selection algorithm in which the probability of estimates being “published” depends nonlinearly on both the sign of the estimated effect and its *P*‐value. They construct three different publication selection regimes which they call “No Publication Bias,” “Medium Publication Bias,” and “Strong Publication Bias.” These are obtained by altering the parameters of the publication selection algorithm. They also simulate four different types of “questionable research practices” (QRPs): (a) optional removal of outliers, (b) optional selection between two dependent variables, (c) optional use of moderators, and (d) optional stopping. Finally, CSG&H also construct experiments in which the simulated MA samples take on four different sizes: 10, 30, 60, and 100.

Table 2 reports the number of experiments for each of the four simulation environments, categorized by number of estimates included in the meta‐analyst's sample (“Sample Size”) and the extent of measured effect heterogeneity (“ *I*^2^”). We calculate *I* ^*2*^ as:

(6) $$I^{2}=\frac{{\hat{\tau }}^{2}}{{\hat{\tau }}^{2}+{\hat{\sigma }}^{2}},$$

where ${\hat{\tau }}^{2}$ is the estimate of effect heterogeneity using the restricted maximum likelihood method, and

(7) $${\hat{\sigma }}^{2}=\frac{\sum w_{i}\left(N-1\right)}{{\left(\sum w_{i}\right)}^{2}-\sum w_{i}^{2}},$$

**TABLE 2:** Number of experiments by sample size and extent of effect heterogeneity

A. Stanley, Doucouliagos, and Ioannidis^1^				
Sample size	Low 𝐼^2^	Moderate 𝐼^2^	High 𝐼^2^	Total
	*I*^2^ ≤ 0.25	0.25 < *I*^2^ ≤ 0.75	0.75 < *I*^2^	
{5,10}	30	27	15	72
20	15	10	11	36
40	15	10	11	36
80	13	12	11	36
{100, 200, 400, 800}	51	49	44	144
Total	124	108	92	324

B. Alinaghi and Reed^2^				
Sample size	Low 𝐼^2^	Moderate 𝐼^2^	High 𝐼^2^	Total
	*I*^2^ ≤ 0.25	0.25 < *I*^2^ ≤ 0.75	0.75 < *I*^2^	
0 < *SS* ≤ 100	0	0	0	0
100 < *SS* ≤ 500	0	0	13	13
500 < *SS*	0	1	22	23
Total	0	1	35	36

C. Bom and Rachinger^3^				
Sample size	Low 𝐼^2^	Moderate 𝐼^2^	High 𝐼^2^	Total
	*I*^2^ ≤ 0.25	0.25 < *I*^2^ ≤ 0.75	0.75 < *I*^2^	
{5, 10}	20	27	65	112
20	5	18	33	56
40	5	17	34	56
80	5	17	34	56
{100, 200, 400, 800}	20	68	136	224
Total	55	147	302	504

D. Carter et al^4^				
Sample size	Low *I*^2^	Moderate *I*^2^	High *I*^2^	Total
	*I*^2^ ≤ 0.25	0.25 < *I*^2^ ≤ 0.75	0.75 < *I*^2^	
10	33	68	7	108
30	29	57	22	108
60	28	54	26	108
100	28	50	30	108
{200, 400, 800}	81	147	96	324
Total	199	376	181	756

*Note:* The table lists the number of experiments for each {sample size, effect heterogeneity} category, by simulation environment. An experiment is defined as a unique set of parameters determining (a) effect size, (b) effect heterogeneity, (c) publication selection, (d) sample size, and (e) (for Carter et al., 2019) questionable research practices (see Appendix 2 in Data S1). Each experiment consists of 3000 simulated meta‐analyses. *I*^2^ measures the share of effect size variance that is due to heterogeneity in true effects. It is based on ${\hat{\tau }}^{2}$, which we, following Carter et al,^4^ estimate using restricted maximum likelihood (REML) [see Equation (6) in the text and the associated discussion].

$w_{i}=1/{\mathrm{SE}}_{i}^{2}$, and *N* is the number of estimates in the meta‐analyst's sample. *I*^2^ takes values between 0 and 100%. *I*^2^ is often interpreted as a measure of the share of effect size variance that is due to heterogeneity in true effects in the population. However, Augusteijn et al.^24^ demonstrate, that it is affected by publication selection. The effect of publication selection can be large, and can either increase or decrease the value of *I*^2^. Our simulations calculate *I*^2^ post‐publication selection. Whether that vitiates the usefulness of *I*^2^ in the selection of estimators is an empirical question.

In order to induce greater overlap in the simulation environments, we added simulations to the SD&I,^1^ B&R,^3^ and CSG&H^4^ experimental designs that allow for larger sample sizes. These are yellow‐highlighted in the table. This resulted in a total of 1620 experiments, where an experiment is defined as a unique set of parameters determining (a) effect size, (b) effect heterogeneity, (c) publication selection, (d) sample size, and (e) (for CSG&H^4^) questionable research practices. This compares favorably with previous studies (see Table 1). Details about the experiments are reported in Appendix 2 in Data S1.

## 4 THE PERFORMANCE MEASURES

We evaluate estimators on three performance measures: (a) Bias, (b) Mean Squared Error (MSE), and (c) 95% Coverage Rates. With respect to bias, the average bias for any given experiment *k* is calculated by

$${\text{Bias}}_{k}=\left(\frac{1}{R_{k}}\right)\sum_{i=1}^{R_{k}}\left({\text{Estimated Effect}}_{\mathrm{ki}}-{\text{True Effect}}_{k}\right),$$

where *R*_*k*_is the total number of iterations for that experiment (typically 3000). Note that *Bias*_*k*_ can be positive or negative. When aggregating over experiments to obtain a summary measure of performance, we calculate the average of absolute values, |*Bias*|= $\left(\frac{1}{R}\right)\sum_{k=1}^{R}\left|{\text{Bias}}_{k}\right|$,, where *R* is the total number of experiments included in the evaluation. “Best estimator” with respect to bias is defined as the estimator with the smallest value of average |*Bias*|.

MSE for a given experiment *k* is calculated by

$${\mathrm{MSE}}_{k}=\left(\frac{1}{R_{k}}\right)\sum_{i=1}^{R_{k}}{\left({\text{Estimated Effect}}_{\mathrm{ki}}-{\text{True Effect}}_{k}\right)}^{2}.$$

When used as a summary measure of performance, it is calculated by *SE*= $\left(\frac{1}{R}\right)\sum_{k=1}^{R}{\mathrm{MSE}}_{k}$. “Best estimator” with respect to MSE is defined as the estimator with the smallest value of MSE.

|Coverage‐0.95| calculates the absolute value of the difference between the coverage rate – the percent of times the 95% confidence interval covers the true effect ‐ and 95%. For example, if the coverage rate in one simulation was 97%, and in another it was 93%, the mean absolute value of the difference would be 2%. “Best estimator” with respect to |Coverage‐0.95| is the estimator that produces values closest to 0.

The previous performance measures apply to all 1620 experiments. The last performance measure, Type I Error Rate, only applies to experiments where the true value of the mean effect equals 0. It measures how often the estimator finds a statistically significant when in fact there is no effect. Good performance on this criterion is represented by values close to 5%.

### 4.1 A caveat about using average performance to assess evaluators

A number of the estimators (3PSM, 4PSM, AK1, AK2, pC, pU, and TF) use maximization procedures to produce their estimates. In some cases, the algorithms do not converge and no estimate is produced. This can cause comparisons of average performance to be misleading. Consider the extreme case where all estimators perform well in simulation environment A but poorly in simulation environment B, but one of the estimators always fails to converge in B. That estimator will appear to be superior based on its average performance. Its superior average performance would reflect differences in experiments, and not differences in actual performance. As an aggregate measure, average performance is suggestive, but conclusions regarding performance should always be based on an inspection of performance at the level of individual experiments.^6^ Section 6 provides a demonstration of this approach.

## 5 RESULTS

### 5.1 Relative performance differs across criteria

Table 3 ranks average performance of the estimators for all 1620 experiments.^7^ Results are separated by performance measure (Bias, MSE, etc.). The purpose of this table is not to demonstrate overall superiority for any given estimator. In addition to the problem of convergence rates discussed above, there is too much heterogeneity in these average numbers for them to be very useful. The main purpose of this table is to note that estimators that dominate on one criterion may perform relatively poorly on another.

**TABLE 3:** Comparison of estimator performance: all experiments

Performance criterion							
|Bias|		MSE		|Coverage‐0.95|^a^		Type I Error^b^	
**EK**	0.076	**WAAP**	0.075	*AK2*	0.172	*AK2*	0.113
**PP**	0.081	**PP**	0.106	3PSM	0.212	**EK**	0.236
*AK2*	0.083	**EK**	0.107	4PSM	0.258	3PSM	0.243
4PSM	0.090	TF	0.110	**PP**	0.290	**PP**	0.267
3PSM	0.101	**AK1**	0.120	**EK**	0.297	4PSM	0.274
**WAAP**	0.109	*AK2*	0.136	**WAAP**	0.310	**WAAP**	0.516
**AK1**	0.132	3PSM	0.140	**AK1**	0.346	**AK1**	0.566
TF	0.140	pU	0.160	TF	0.396	**TF**	0.586
**RE**	0.216	4PSM	0.163	**RE**	0.512	pU	0.589
pU	0.229	**RE**	0.195	pU	0.578	**RE**	0.640
pC	0.333	pC	0.608	pC	NA	pC	NA

*Note:* The values in the table represent the average values of the respective performance measures across all 1620 experiments for the first three columns. The last column only reports results for those experiments where the true mean effect = 0. The three “best” performing estimators on the dimensions of Bias, MSE, and Coverage rates/Type I Error (EK, WAAP, and AK2) are color‐coded to facilitate comparison across performance measures. Estimators: 3PSM/4PSM = Three‐Parameter/Four‐Parameter Selection Models AK1 = Andrews and Kasy's^16^ “symmetric selection” model AK2 = Andrews and Kasy's^16^ “asymmetric selection” selection EK = Bom and Rachinger's^3^ Endogenous Kink estimator pC = p‐curve pU = p‐uniform PP = PET‐PEESE (Stanley and Doucouliagos^22^) RE = Random Effects TF = Trim and Fill WAAP = Stanley et al's^1^ Weighted Average of the Adequately Powered‐WLS hybrid estimator.

^a^ This column reports the average, absolute value of the difference between (a) the percent of times the 95% confidence interval contains the true mean value and (b) 95%.

^b^ This column reports the percentage of false positives when the true mean effect = 0; that is, the percent of times an estimate is statistically significant when there is no true effect.

For example, on the dimension of bias, Bom and Rachinger's^3^ Endogenous Kink (EK) estimator produces the lowest overall, mean absolute bias (“|Bias|”). However, it is dominated by Stanley, Doucouliagos, and Ioannidis's^1^ WAAP estimator when it comes to mean squared error (“MSE”); and Andrews and Kasy's^16^ “asymmetric selection” estimator (AK2) with respect to |Coverage‐0.95| and Type I Error rates. The table color‐codes the three estimators with best average performance on the respective criteria to facilitate comparison. It highlights that superior performance on one dimension does not guarantee superior performance on another. As a result, when choosing an estimator, meta‐analysts should prioritize which performance measure is of most importance.

Table 3 also highlights the poor performance of all the estimators when it comes to coverage and Type I error rates. While AK2 may be the “best” performing estimator on these dimensions, the mean absolute deviation between the coverage rate and 95% is 17%. The mean Type I error rate is 11%, compared to an expectation of 5%. The other estimators perform much worse. Most of the estimators have coverage rates below 70% and Type I error rates larger than 25%. This should cause researchers to question the reliability of any hypothesis testing about effect sizes that is performed in meta‐analyses that use these estimators.

### 5.2 Relative performance differs across simulation environments

We next focus on the sensitivity of results to simulation environment. Table 4 collects the results from all 1620 experiments and breaks them out according to each of the four simulation environments and each of the four performance criteria. In both panels, we are looking for consistency in relative performance across simulation environments.

**TABLE 4:** Comparison of Estimator Performance across Simulation Environments

A. |Bias|							
*SD&I* ^1^		*A&R* ^2^		*B&R* ^3^		*CSG&H* ^4^	
*AK2* ^*a*^	0.031	*AK2*	0.200	*AK2*	0.071	**PP**	0.058
**3PSM** ^**a**^	0.036	**EK**	0.213	**EK**	0.089	**WAAP**	0.062
**4PSM**	0.040	**PP**	0.256	**4PSM**	0.099	**AK1**	0.064
**PP**	0.050	**WAAP**	0.263	**PP**	0.124	**EK**	0.071
**EK**	0.053	**TF**	0.284	**3PSM**	0.147	*3PSM*	0.080
**AK1**	0.060	**4PSM**	0.298	**WAAP**	0.187	TF	0.091
**WAAP**	0.083	**AK1**	0.390	**TF**	0.238	*4PSM*	0.095
**TF**	0.088	**3PSM**	0.468	**AK1**	0.262	**pU**	0.105
**RE**	0.107	**RE**	0.550	**RE**	0.361	*AK2*	0.107
pU^a^	0.146	**pC**	1.530	pU	0.373	**pC**	0.114
pC	0.420	**pU**	1.556	pC	0.521	**RE**	0.150

B. MSE							
*SD&I* ^1^		*A&R* ^2^		*B&R* ^3^		*CSG&H* ^4^	
*AK2*	0.013	*AK2*	0.229	**WAAP**	0.171	**AK1**	0.011
**3PSM**	0.013	**TF**	0.244	pU	0.184	**WAAP**	0.016
**AK1**	0.016	**4PSM**	0.318	**EK**	0.250	*3PSM*	0.021
**WAAP**	0.024	**AK1**	0.363	**PP**	0.262	TF	0.021
**TF**	0.024	**WAAP**	0.423	**TF**	0.289	**PP**	0.021
**PP**	0.024	**PP**	0.456	*AK2*	0.324	**EK**	0.025
**EK**	0.026	**3PSM**	0.468	**AK1**	0.333	**pU**	0.025
**RE**	0.033	**RE**	0.484	**4PSM**	0.366	*4PSM*	0.028
pU	0.049	**EK**	0.567	**3PSM**	0.376	*AK2*	0.036
**4PSM**	0.144	**pC**	3.518	**RE**	0.502	**RE**	0.045
pC	1.209	**pU**	3.626	pC	0.836	**pC**	0.060

C. |Coverage‐0.95|^b^							
*SD&I* ^1^		*A&R* ^2^		*B&R* ^3^		*CSG&H* ^4^	
*AK2*	0.104	*AK2*	0.268	*AK2*	0.040	**WAAP**	0.285
**3PSM**	0.155	**4PSM**	0.367	**4PSM**	0.071	*AK2*	0.298
**PP**	0.183	**AK1**	0.542	**3PSM**	0.076	*3PSM*	0.308
**EK**	0.196	**TF**	0.557	**EK**	0.092	**AK1**	0.364
**4PSM**	0.220	**WAAP**	0.291	**PP**	0.136	*4PSM*	0.394
**AK1**	0.291	**3PSM**	0.600	**WAAP**	0.317	**PP**	0.419
**WAAP**	0.324	**EK**	0.702	**TF**	0.339	TF	0.431
**TF**	0.385	**PP**	0.712	**AK1**	0.340	**pU**	0.454
**RE**	0.421	**RE**	0.818	**RE**	0.416	**EK**	0.459
pU	0.459	**pU**	0.924	pU	0.808	**RE**	0.601
pC	NA	**pC**	NA	pC	NA	**pC**	NA

D. Type 1 Error^c^							
*SD&I* ^1^		*A&R* ^2^		*B&R* ^3^		*CSG&H* ^4^	
*AK2*	0.086	*AK2*	0.300	*AK2*	0.029	*AK2*	0.254
**EK**	0.212	**4PSM**	0.597	**4PSM**	0.083	**EK**	0.400
**PP**	0.249	AK1	0.629	**3PSM**	0.096	*3PSM*	0.413
3PSM	0.257	**RE**	0.630	**EK**	0.118	**PP**	0.419
**4PSM**	0.332	**TF**	0.637	**PP**	0.156	pU	0.436
pU	0.526	**WLS**	0.719	**WLS**	0.497	*4PSM*	0.474
AK1	0.526	**PP**	0.791	**TF**	0.518	**WLS**	0.498
**WLS**	0.562	**EK**	0.797	**AK1**	0.539	**AK1**	0.632
**RE**	0.612	3PSM	0.919	**RE**	0.602	TF	0.655
**TF**	0.613	pU	1.000	pU	0.721	**RE**	0.712
pC	NA	pC	NA	pC	NA	pC	NA

*Note:* The four panels rank the performance of the 11 estimators on the basis of their average Bias, MSE, |Coverage‐0.95|, and Type I Error performance, disaggregated by simulation environment. Estimators are ranked from “best” (least Bias, smallest MSE, etc.) to worst. Values in the tables are the average values for the respective performance measures and simulation environments. In each panel the best and second best performing estimators in the SD&I environments are color‐coded brown and gray, respectively. This allows one to track their relative performance across the remaining three simulation environments.

^a^ It is important to note that the maximization procedures that underlie some of the estimators do not always converge. Averages across estimators will not be comparable if they average across different experiments due to lack of convergence. To indicate this in the table, we indicate three types of convergence behaviour. Boldfaced estimators indicate a convergence rate of 99% or higher (eg, **AK1)**. Conventional, non‐boldfaced type indicates that the estimator converged between 90%‐99% of the time (eg, AK1). Italicized estimators indicate that convergence rates were lower than 90% (eg, *AK1*).

^b^ This panel reports the average, absolute value of the difference between (i) the percent of times the 95% confidence interval contains the true mean value and (ii) 95%.

^c^ This panel reports the percentage of false positives when the true mean effect = 0; that is, the percent of times an estimate is statistically significant when there is no true effect.

In Panel A, which reports performance with respect to bias, simulations for three of the four simulation environments show that the AK2 estimator has best average performance, as measured by smallest mean absolute bias. However, in the CSG&H simulations, the AK2 estimator ranks 9th of 11. The 3PSM estimator ranks second in the SD&I simulations with respect to mean absolute bias, but 8th in A&R's, 5th in B&R's, and 5th in CSG&H's simulations. These inconsistencies are not unusual. Panel B ranks average performance of the estimators with respect to MSE. The AK2 estimator ranks 1st in the SD&I and A&R simulations, but 6th in B&R's simulations, and 9th in CSG&H's. Other inconsistencies across simulation environments are easily found in Panels C and D.

Table 1 demonstrated that it was difficult to draw guidance from previous studies about which estimator to use because there was little overlap in the estimators being compared. Table 4 identifies a more critical issue. Even when the same estimators are being compared, one can obtain different results depending on which simulation design is being used. This raises the question: what are the factors responsible for these differences? A full treatment of the question lies beyond the scope of this study. However, we undertake an initial effort at answering this question by focusing on two features of the simulation designs: number of estimates in the simulated MA samples (“sample size”), and the extent of effect size heterogeneity, as measured by *I* ^*2*^. Table 2 highlights that the different simulation environments differ on these dimensions. If these two features systematically affect estimator performance, then differences in the combinations of sample size and effect heterogeneity would provide at least a partial explanation for the differences in average performance across simulation environments.

### 5.3 The influence of sample size and effect heterogeneity on relative estimator performance

It is well‐known that estimator performance generally declines as effect heterogeneity increases and improves as the meta‐analyst's sample size gets larger (Moreno et al.,^8^; Stanley^12^). Less well‐known is that relative estimator performance is also affected by these factors. In this section we demonstrate both phenomena. We use the results from the CSG&H simulations to estimate the following regressions for each of the 11 estimators (*j*):

(8a) $${\text{Bias}}_{\mathrm{ij}}=\beta_{0}+\beta_{\text{SampleSize}}∙{\left(\text{SampleSize}\right)}_{\mathrm{ij}}+\beta_{I^{2}}∙{\left(I^{2}\right)}_{\mathrm{ij}}+\varepsilon_{\mathrm{ij}},$$

and

(8b) $${\mathrm{MSE}}_{\mathrm{ij}}=\beta_{0}+\beta_{\text{SampleSize}}∙{\left(\text{SampleSize}\right)}_{\mathrm{ij}}+\beta_{I^{2}}∙{\left(I^{2}\right)}_{\mathrm{ij}}+\varepsilon_{\mathrm{ij}}.$$

Regressions were estimated using OLS with bootstrapped *t*‐statistics to obtain *P*‐values. Each regression used the Bias/MSE results for a given estimator *j*. The respective samples were constructed from the individual results of the 756 experiments in the CSG&H simulations (see Panel D of Table 2).

Table 5 presents the results. They provide strong evidence that Bias and MSE increase as effect heterogeneity (*I*^2^) increases. With only one exception, the coefficient on the *I*^2^ term is positive and significant in both the Bias and MSE regressions for each of the 11 estimators. The exception is the coefficient for *I*^2^ in the MSE regression for the p‐curve estimator (pC). Sample size is also strongly associated with MSE performance. Sample size is negatively and significantly associated with MSE for each of the 11 estimators. The evidence for sample size affecting bias is not as strong. Still, 9 of the 11 estimated coefficients are negative, with 5 of 11 negative and significant at the 5% level.

**TABLE 5:** Sample size and effect heterogeneity as determinants of absolute estimator performance: CSG&H^4^ simulation environment

Estimator	Bias		MSE	
	*β*_*SampleSize*_	$\beta_{I^{2}}$	*β*_*SampleSize*_	$\beta_{I^{2}}$
***AK1***	−0.0143* (0.0074)	0.1147*** (0.0068)	−0.0101*** (0.0018)	0.0240*** (0.0018)
***4PSM***	0.0112 (0.0116)	0.2214*** (0.0098)	−0.0160*** (0.0045)	0.0812*** (0.0040)
***3PSM***	0.0029 (0.0101)	0.1624*** (0.0088)	−0.0156*** (0.0034)	0.0536*** (0.0030)
***WAAP***	−0.0366*** (0.0091)	0.1163*** (0.0078)	−0.0235*** (0.0027)	0.0344*** (0.0024)
***TF***	−0.0150 (0.0121)	0.1555*** (0.0093)	−0.0121*** (0.0042)	0.0413*** (0.0033)
***AK2***	−0.0355** (0.0168)	0.1883*** (0.0122)	−0.0458*** (0.0099)	0.0676*** (0.0075)
***PP***	−0.0206*** (0.0069)	0.0868*** (0.0060)	−0.0443*** (0.0040)	0.0468*** (0.0037)
***RE***	−0.0222 (0.0178)	0.2180*** (0.0151)	−0.0139** (0.0083)	0.0927*** (0.0071)
***EK***	−0.0286*** (0.0058)	0.0125*** (0.0058)	−0.055*** (0.0041)	0.0399*** (0.0039)
***pU***	−0.0180 (0.0124)	0.1352*** (0.0121)	−0.0182*** (0.0050)	0.0575*** (0.0053)
***pC***	−0.0403*** (0.0151)	0.1360*** (0.0150)	−0.1140*** (0.0302)	−0.0025 (0.0318)

*Note:* The table reports the results of estimating Equations (8a) and (8b) in the text. Regressions were estimated using OLS with bootstrapped *t*‐statistics to obtain *p*‐values. Each regression used the Bias/MSE results for a given estimator *j*. The respective samples were constructed from the individual results of the 756 experiments in the Carter, Schönbrodt, Gervais, and Hilgard^4^ simulations. Bootstrap SEs are reported in parentheses. When estimating the model we use Sample size/1000. This transformation increases the size of ***β***_***SampleSize***_ by a factor of 1000, but leaves economic and statistical significance unchanged.

While Table 5 documents changes in absolute estimator performance, Table 6 presents evidence of changes in relative performance. Once again we use the CSG&H simulation results and focus on bias and MSE. We divide the 756 CSG&H experiments into 21 separate cells depending on sample size (10, 30, 60, 100, 200, 400, 800) and effect heterogeneity (*I*^2^ ≤ 0.25, 0.25 < *I*^2^ ≤ 0.75, 0.75 < *I*^2^). Panel D of Table 2 reports the number of experiments for each sample size/ *I*^2^ cell.

**TABLE 6:** The relationship between relative estimator performance, sample size, and *I* ^*2*^: CSG&H^4^ simulation environment

A. Sample size = 10											
*Bias*						*MSE*					
*Low I* ^2^		*Moderate I* ^2^		*High I* ^2^		*Low I* ^2^		*Moderate I* ^2^		*High I* ^2^	
AK1^a^	0.028	4PSM	0.071	**AK1**	0.097	AK1	0.006	**AK1**	0.027	**AK1**	0.027
4PSM	0.033	3PSM	0.074	**WAAP**	0.104	3PSM	0.007	pU	0.042	**TF**	0.045
3PSM	0.035	**PP**	0.087	**TF**	0.110	4PSM	0.008	TF	0.043	**WAAP**	0.057
**WAAP** ^**a**^	0.040	**AK1**	0.088	*AK2*	0.119	TF	0.010	3PSM	0.043	**3PSM**	0.069
TF	0.042	**EK**	0.098	**3PSM**	0.146	**WAAP**	0.010	**WAAP**	0.046	**RE**	0.075
*AK2* ^a^	0.047	pU	0.107	**EK**	0.153	*AK2*	0.010	4PSM	0.049	*AK2*	0.078
**PP**	0.063	**WAAP**	0.112	**PP**	0.160	**RE**	0.018	**RE**	0.068	**4PSM**	0.093
**RE**	0.082	TF	0.127	**4PSM**	0.177	**PP**	0.021	**PP**	0.081	pU	0.114
pU	0.090	pC	0.147	**RE**	0.179	pU	0.023	**EK**	0.092	pC	0.164
**EK**	0.101	*AK2*	0.160	pU	0.253	**EK**	0.030	*AK2*	0.102	**PP**	0.209
pC	0.150	**RE**	0.188	pC	0.270	pC	0.278	pC	0.203	**EK**	0.220

B. Sample size = 30											
*Bias*						*MSE*					
*Low I* ^2^		*Moderate I* ^2^		*High I* ^2^		*Low I* ^2^		*Moderate I* ^2^		*High I* ^2^	
**WAAP**	0.012	**PP**	0.048	**EK**	0.094	**WAAP**	0.002	**AK1**	0.011	**AK1**	0.026
**AK1**	0.019	**AK1**	0.068	**PP**	0.106	**AK1**	0.002	**pU**	0.015	**TF**	0.041
TF	0.020	**EK**	0.071	**AK1**	0.115	TF	0.002	3PSM	0.020	**WAAP**	0.045
*3PSM*	0.026	3PSM	0.074	**WAAP**	0.116	*3PSM*	0.003	**PP**	0.020	**3PSM**	0.053
*4PSM*	0.026	**pU**	0.076	**TF**	0.144	*4PSM*	0.003	**WAAP**	0.020	**PP**	0.071
*AK2*	0.028	**WAAP**	0.078	**3PSM**	0.145	**PP**	0.004	4PSM	0.024	**EK**	0.077
**PP**	0.029	4PSM	0.079	**4PSM**	0.190	*AK2*	0.004	TF	0.024	**pU**	0.077
**RE**	0.049	**pC**	0.083	**RE**	0.202	**RE**	0.005	**EK**	0.030	**4PSM**	0.078
**EK**	0.073	TF	0.102	*AK2*	0.217	**EK**	0.011	*AK2*	0.036	**pC**	0.081
pU	0.081	*AK2*	0.113	**pU**	0.218	pU	0.014	**pC**	0.049	**RE**	0.084
pC	0.094	**RE**	0.181	**pC**	0.224	pC	0.077	**RE**	0.052	*AK2*	0.096

C. Sample size = 60											
*Bias*						*MSE*					
*Low I* ^2^		*Moderate I* ^2^		*High I* ^2^		*Low I* ^2^		*Moderate I* ^2^		*High I* ^2^	
**WAAP**	0.010	**PP**	0.050	**EK**	0.081	**WAAP**	0.001	**AK1**	0.009	**AK1**	0.021
TF	0.016	**EK**	0.065	**PP**	0.089	TF	0.001	**pU**	0.012	**WAAP**	0.033
**AK1**	0.017	**AK1**	0.066	**WAAP**	0.104	**AK1**	0.001	**PP**	0.012	**TF**	0.034
*3PSM*	0.022	**WAAP**	0.066	**AK1**	0.107	*3PSM*	0.002	**WAAP**	0.014	**PP**	0.040
*4PSM*	0.023	**pU**	0.073	**3PSM**	0.137	**PP**	0.002	**EK**	0.018	**3PSM**	0.041
**PP**	0.024	**pC**	0.073	**TF**	0.138	*4PSM*	0.002	3PSM	0.018	**EK**	0.044
*AK2*	0.026	3PSM	0.084	*AK2*	0.163	*AK2*	0.003	**pC**	0.018	*AK2*	0.050
**RE**	0.042	4PSM	0.090	**4PSM**	0.189	**RE**	0.004	4PSM	0.022	**pU**	0.065
**EK**	0.063	TF	0.102	**RE**	0.201	**EK**	0.007	TF	0.022	**4PSM**	0.065
**pU**	0.074	*AK2*	0.118	**pU**	0.205	**pU**	0.010	*AK2*	0.035	**pC**	0.068
**pC**	0.081	**RE**	0.180	**pC**	0.213	**pC**	0.049	**RE**	0.051	**RE**	0.075

D. Sample size = 100											
*Bias*						*MSE*					
*Low I* ^2^		*Moderate I* ^2^		*High I* ^2^		*Low I* ^2^		*Moderate I* ^2^		*High I* ^2^	
**WAAP**	0.009	**PP**	0.049	**EK**	0.073	**WAAP**	0.001	**AK1**	0.007	**AK1**	0.020
TF	0.015	**WAAP**	0.055	**PP**	0.086	TF	0.001	**PP**	0.009	**WAAP**	0.027
**AK1**	0.017	**AK1**	0.061	**WAAP**	0.104	**AK1**	0.001	**pC**	0.009	**PP**	0.028
*AK2*	0.021	**EK**	0.064	**AK1**	0.110	**PP**	0.001	**pU**	0.009	**EK**	0.028
*3PSM*	0.021	**pC**	0.066	**3PSM**	0.131	*3PSM*	0.001	**WAAP**	0.010	**3PSM**	0.034
**PP**	0.022	**pU**	0.068	*AK2*	0.148	*4PSM*	0.002	**EK**	0.013	**TF**	0.035
*4PSM*	0.022	3PSM	0.089	**TF**	0.149	*AK2*	0.002	3PSM	0.018	*AK2*	0.041
**RE**	0.041	TF	0.094	**4PSM**	0.179	**RE**	0.003	TF	0.019	**4PSM**	0.056
**EK**	0.060	4PSM	0.097	**pU**	0.196	**EK**	0.006	4PSM	0.021	**pU**	0.058
**pU**	0.071	*AK2*	0.108	**pC**	0.204	**pU**	0.009	*AK2*	0.026	**pC**	0.062
**pC**	0.074	**RE**	0.168	**RE**	0.218	**pC**	0.030	**RE**	0.046	**RE**	0.079

E. Sample size = 200											
*Bias*						*MSE*					
*Low I* ^2^		*Moderate I* ^2^		*High I* ^2^		*Low I* ^2^		*Moderate I* ^2^		*High I* ^2^	
**WAAP**	0.008	**PP**	0.048	**EK**	0.072	TF	0.001	**AK1**	0.006	**EK**	0.018
TF	0.013	**WAAP**	0.052	**PP**	0.089	**WAAP**	0.001	**PP**	0.007	**AK1**	0.019
**AK1**	0.016	**AK1**	0.060	**WAAP**	0.097	**AK1**	0.001	**WAAP**	0.008	**PP**	0.021
*AK2*	0.020	**EK**	0.063	**AK1**	0.109	**PP**	0.001	**pC**	0.008	**WAAP**	0.022
*3PSM*	0.020	**pC**	0.067	**3PSM**	0.132	*3PSM*	0.001	**pU**	0.009	**3PSM**	0.033
*4PSM*	0.021	**pU**	0.068	*AK2*	0.144	*4PSM*	0.001	**EK**	0.009	**TF**	0.034
**PP**	0.021	*3PSM*	0.091	**TF**	0.151	*AK2*	0.001	*3PSM*	0.017	*AK2*	0.036
**RE**	0.036	TF	0.095	**4PSM**	0.185	**RE**	0.002	TF	0.019	**4PSM**	0.055
**EK**	0.057	*4PSM*	0.100	**pU**	0.196	**EK**	0.005	*4PSM*	0.021	**pU**	0.056
**pC**	0.063	*AK2*	0.121	**pC**	0.207	**pC**	0.007	*AK2*	0.028	**pC**	0.061
**pU**	0.067	**RE**	0.167	**RE**	0.218	**pU**	0.007	**RE**	0.045	**RE**	0.078

F. Sample size = 400											
*Bias*						*MSE*					
*Low I* ^2^		*Moderate I* ^2^		*High I* ^2^		*Low I* ^2^		*Moderate I* ^2^		*High I* ^2^	
**WAAP**	0.008	**PP**	0.046	**EK**	0.070	TF	0.000	**PP**	0.005	**EK**	0.013
TF	0.013	**WAAP**	0.048	**PP**	0.091	**WAAP**	0.000	**AK1**	0.006	**AK1**	0.018
**AK1**	0.016	**AK1**	0.059	**WAAP**	0.097	**AK1**	0.001	**WAAP**	0.006	**PP**	0.018
*3PSM*	0.020	**EK**	0.061	**AK1**	0.107	**PP**	0.001	**EK**	0.007	**WAAP**	0.020
*4PSM*	0.020	**pC**	0.064	**3PSM**	0.139	*3PSM*	0.001	**pC**	0.007	**3PSM**	0.033
**PP**	0.021	**pU**	0.066	**TF**	0.150	*4PSM*	0.001	**pU**	0.008	**TF**	0.033
*AK2*	0.021	*3PSM*	0.087	*AK2*	0.158	*AK2*	0.001	*3PSM*	0.015	*AK2*	0.039
**RE**	0.036	*4PSM*	0.093	**pU**	0.187	**RE**	0.002	*4PSM*	0.017	**pU**	0.052
**EK**	0.056	TF	0.093	**4PSM**	0.193	**EK**	0.004	TF	0.018	**4PSM**	0.055
**pC**	0.061	*AK2*	0.115	**pC**	0.200	**pC**	0.006	*AK2*	0.026	**pC**	0.057
**pU**	0.065	**RE**	0.161	**RE**	0.222	**pU**	0.007	**RE**	0.042	**RE**	0.078

G. Sample size = 800											
*Bias*						*MSE*					
*Low I* ^2^		*Moderate I* ^2^		*High I* ^2^		*Low I* ^2^		*Moderate I* ^2^		*High I* ^2^	
**WAAP**	0.007	**PP**	0.046	**EK**	0.070	**WAAP**	0.000	**PP**	0.005	**EK**	0.010
TF	0.013	**WAAP**	0.047	**PP**	0.093	TF	0.000	**EK**	0.006	**PP**	0.017
**AK1**	0.015	**AK1**	0.058	**WAAP**	0.097	**AK1**	0.001	**WAAP**	0.006	**AK1**	0.017
*4PSM*	0.019	**EK**	0.060	**AK1**	0.107	**PP**	0.001	**AK1**	0.006	**WAAP**	0.018
*3PSM*	0.020	**pC**	0.064	**3PSM**	0.140	*3PSM*	0.001	**pC**	0.007	**3PSM**	0.032
**PP**	0.020	**pU**	0.066	**TF**	0.150	*4PSM*	0.001	**pU**	0.007	**TF**	0.033
*AK2*	0.021	*3PSM*	0.087	*AK2*	0.162	*AK2*	0.001	*3PSM*	0.015	*AK2*	0.040
**RE**	0.036	*4PSM*	0.093	**pU**	0.187	**RE**	0.002	*4PSM*	0.017	**pU**	0.052
**EK**	0.055	TF	0.094	**4PSM**	0.195	**EK**	0.004	TF	0.018	**4PSM**	0.056
**pC**	0.060	*AK2*	0.101	**pC**	0.201	**pC**	0.006	*AK2*	0.020	**pC**	0.058
**pU**	0.064	**RE**	0.161	**RE**	0.222	**pU**	0.006	**RE**	0.042	**RE**	0.078

*Note:* The panels above rank the performance of the 11 estimators on the basis of their average Bias and MSE performance, disaggregated by {sample size, effect heterogeneity} categories. Estimators are ranked from “best” (least Bias, smallest MSE) to worst. Values in the tables are the average values for the respective performance measures and {sample size, effect heterogeneity} categories. For both Bias and MSE, the top two estimators in the cell for smallest sample size (10) and effect heterogeneity (low *I* ^2^) are identified by color‐coding. For Bias, these are the AK1 and 4PSM estimators. For MSE, they are AK1 and 3PSM. The relative position of these estimators are then tracked as sample size and effect heterogeneity increases.

^a^ It is important to note that the maximization procedures that underlie some of the estimators do not always converge. Averages across estimators will not be comparable if they average across different experiments due to lack of convergence. To indicate this in the table, we indicate three types of convergence behaviour. Boldfaced estimators indicate a convergence rate of 99% or higher (eg, **AK1)**. Conventional, non‐boldfaced type indicates that the estimator converged between 90%–99% of the time (eg, AK1). Italicized estimators indicate that convergence rates were lower than 90% (eg, *AK1*).

For both Bias and MSE, we identify the top two estimators in the cell for smallest sample size (10) and effect heterogeneity (low *I* ^*2*^). For Bias, these are the AK1 and 4PSM estimators. For MSE, they are AK1 and 3PSM. We then track the relative position of these estimators as sample size and effect heterogeneity increases. The respective estimators are color‐coded to facilitate tracking across cells.

Table 6 clearly reveals that there is substantial movement in the relative rankings of average estimator performance as sample size and effect heterogeneity change. For the sake of brevity, we only report results for Bias and MSE.^8^ In some cases, the change in relative ranking is dramatic. When sample size = 10, the 4PSM estimator ranks 2nd and 1st on Bias, respectively, for low and moderate effect heterogeneity. It falls to 9th when effect heterogeneity is high. In other cases, relative performance is relatively stable: Across all sample sizes, AK1 is either ranked 1st or 2nd in terms of smallest average MSE.

The table demonstrates two things. It underscores a point made previously that no estimator dominates in all research settings. However, it also suggests that there may be circumstances where one estimator is generally preferred. For example, if a researcher is interested in estimator efficiency and works in an area where effect heterogeneity is expected to be high, *and* if the researcher is convinced that the CSG&H simulation environment captures the key elements of their research situation, then Table 6 suggests that AK1 may be the best estimator for their analysis. However, the Table 6 results are based on average performance within a given {sample size, effect heterogeneity} cell. As demonstrated previously, averages can conceal much variation. The next section illustrates how further investigation can lead to a more definitive conclusion regarding “best” estimator.

## 6 AN EXAMPLE OF HOW SIMULATION EXPERIMENTS CAN GUIDE THE SELECTION OF A “BEST” ESTIMATOR

Previous sections demonstrated that there is no superior estimator for all research situations. “Best” is conditional on performance measure, and depends on observable characteristics of the meta‐analyst's sample such as sample size and effect heterogeneity. It also can depend on unobservable characteristics such as the type of publication selection (statistical significance, correct sign, both), the extent of publication selection, and other factors such as assorted questionable research practices (QRPs). By conditioning on observables and investigating performance over unobservables, one can study the relative performance of estimators and use the results to guide estimator selection for use in a given research situation. This section demonstrates how this can be done.

Suppose a meta‐analyst is studying the empirical literature on a given “effect,” measured by Cohen's *d*. They collect a sample of 100 estimates. Initial analysis indicates a high degree of effect heterogeneity (*I*^2^ > 0.75). While they are unsure whether publication selection is a problem, if it does exist, they believe selection would depend on both correct sign and statistical significance. Looking over the alternatives, it is their experienced judgment that the CSG&H simulation environment best captures the salient aspects of their research situation. However, they do not have strong priors about the size of the effect, the severity of publication selection, nor the extent of QRPs. While they would like to have an estimator that minimized bias, produced accurate coverage rates, and provided reliable tests of significance, their main priority is choosing an estimator that is efficient. We show how simulation results can be used to guide that selection.

Table 7 reports the individual experimental results for sample size = 100/ High *I*^2^. There are a total of 30 experimental results (cf. Table 2), covering a wide range of effect sizes {0, 0.2, 0.5, 0.8}, severities of publication selection {No, Medium, Strong}, and QRP behaviors {None, Medium, High} (see Appendix 2 in Data S1). We suppose the meta‐analyst is interested in not just average MSE performance, but also the variation of MSE across situations. Since they do not know which of the respective experiments best represents their research situation, they want to avoid an estimator that occasionally produces a bad result, even if it does well on average.

**TABLE 7:** Comparison of MSE performance: Sample size = 100, high *I,* ^2^ CSG&H^4^ simulation environment

*{Effect Size, I* ^*2*^ *, QRP, Publication Selection}*	*Estimators*										
	*TF*	*pC*	*pU*	*RE*	*3PSM*	*4PSM*	*AK1*	*AK2*	*WAAP*	*PP*	*EK*
{0, 0.822, None, No}	0.005	0.207	0.203	0.002	0.004	0.008	0.002	0.012	0.007	0.019	0.025
{0.2, 0.821, None, No}	0.006	0.110	0.106	0.002	0.004	0.008	0.002	0.012	0.017	0.019	0.025
{0.5, 0.818, None, No}	0.007	0.032	0.029	0.002	0.004	0.008	0.003	0.011	0.014	0.012	0.027
{0.8, 0.810, None, No}	0.010	0.004	0.003	0.002	0.005	0.008	0.004	0.009	0.010	0.013	0.029
{0, 0.864, Med, No}	0.004	0.101	0.096	0.003	0.020	0.028	0.001	0.025	0.005	0.030	0.034
{0.2, 0.856, Med, No}	0.006	0.046	0.040	0.003	0.032	0.037	0.003	0.034	0.016	0.035	0.039
{0.5, 0.835, Med, No}	0.012	0.008	0.006	0.003	0.048	0.066	0.012	0.079	0.015	0.027	0.043
{0.8, 0.805, Med, No}	0.019	0.003	0.004	0.003	0.051	0.079	0.021	0.083	0.010	0.016	0.039
{0, 0.879, High, No}	0.003	0.070	0.065	0.004	0.040	0.050	0.001	0.043	0.005	0.042	0.045
{0.2, 0.869, High, No}	0.006	0.029	0.024	0.005	0.063	0.065	0.004	0.055	0.015	0.050	0.052
{0.5, 0.837, High, No}	0.010	0.005	0.003	0.006	0.096	0.109	0.018	0.120	0.013	0.034	0.052
{0.8, 0.803, High, No}	0.020	0.005	0.007	0.004	0.109	0.142	0.031	0.142	0.011	0.019	0.047
{0, 0.769, None, Med}	0.022	0.056	0.055	0.053	0.009	0.020	0.021	0.009	0.020	0.014	0.010
{0, 0.763, Med, Med}	0.047	0.009	0.009	0.100	0.006	0.018	0.014	0.009	0.020	0.011	0.008
{0, 0.757, High, Med}	0.061	0.003	0.003	0.125	0.010	0.012	0.013	0.005	0.021	0.013	0.010
{0, 0.920, None, Med}	0.031	0.201	0.197	0.103	0.006	0.086	0.041	0.023	0.040	0.030	0.027
{0.2, 0.859, None, Med}	0.034	0.107	0.103	0.106	0.009	0.063	0.027	0.022	0.033	0.034	0.022
{0.5, 0.785, None, Med}	0.012	0.030	0.028	0.058	0.007	0.021	0.011	0.018	0.016	0.012	0.017
{0.8, 0.774, None, Med}	0.003	0.003	0.003	0.023	0.004	0.007	0.003	0.012	0.007	0.008	0.026
{0, 0.908, Med, Med}	0.050	0.100	0.090	0.136	0.072	0.151	0.029	0.048	0.040	0.027	0.024
{0.2, 0.829, Med, Med}	0.036	0.045	0.038	0.119	0.060	0.146	0.009	0.060	0.030	0.030	0.021
{0, 0.901, High, Med}	0.060	0.069	0.060	0.153	0.108	0.136	0.026	0.042	0.042	0.030	0.027
{0.2, 0.816, High, Med}	0.038	0.029	0.022	0.124	0.111	0.145	0.006	0.067	0.030	0.032	0.022
{0, 0.755, None, Strong}	0.071	0.056	0.056	0.133	0.010	0.009	0.036	‐^a^	0.033	0.030	0.011
{0, 0.895, None, Strong}	0.116	0.202	0.196	0.255	0.019	0.101	0.081	‐^a^	0.087	0.074	0.043
{0.2, 0.807, None, Strong}	0.080	0.108	0.104	0.185	0.018	0.043	0.050	‐^a^	0.064	0.053	0.023
{0.5, 0.759, None, Strong}	0.019	0.030	0.028	0.083	0.009	0.013	0.014	‐^a^	0.023	0.015	0.014
{0.8, 0.768, None, Strong}	0.002	0.003	0.003	0.031	0.005	0.006	0.003	‐^a^	0.008	0.008	0.027
{0, 0.843, Med, Strong}	0.130	0.101	0.090	0.271	0.051	0.053	0.056	‐^a^	0.081	0.060	0.034
{0, 0.823, High, Strong}	0.135	0.071	0.060	0.277	0.041	0.041	0.051	‐^a^	0.080	0.057	0.031
*Average MSE =*	0.035	0.062	0.058	0.079	0.034	0.056	0.020	0.041	0.027	0.028	0.028
*(Smallest, Largest) =*	(0.002,0.135)	(0.003,0.207)	(0.003,0.203)	(0.002,0.277)	(0.004,0.111)	(0.006,0.151)	(0.001,0.081)	(0.005,0.142)	(0.005,0.087)	(0.008,0.074)	(0.008,0.052)

*Note:* This table reports estimator MSE performance results for the 30 experiments included within the {sample size = 100, high *I* ^2^} category of the CSG&H^4^ simulations. The estimators are described in Section 2 of the text. The first column gives details about the individual experiment (cf. the bottom panel in Appendix 2 in Data S1). Each cell represents results for a single experiment consisting of 3000 simulated meta‐analyses. Each simulated meta‐analysis produces a single estimate of the mean population effect. The numbers in the table are the averaged mean squared error (MSE) value for the 3000 simulated meta‐analyses for that estimator and experiment. The last two rows of each panel report the overall average MSE, followed by the smallest and largest (average) MSE values over the 30 experiments. Yellow‐highlighted cells in the upper panel of the table identify the smallest (average) MSE for each experiment. The yellow‐highlighted cell in the bottom panel of the table identifies the estimator (AK1) with the lowest overall, averaged MSE value. The blue‐highlighted cells identify estimators that are close to AK1 in terms of overall performance.

^a^ Indicates that all estimates failed to converge for that experiment.

The top part of the table reports the individual MSE experimental results. We yellow‐highlight the minimum MSE value in each experiment. Of the 11 estimators, all but two of them (WAAP and PP) are “best” in at least one experiment. This again highlights the fact that no estimator is best in all research situations. To assist in processing the large amount of information in the table, we report average performance for the 30 experiments, along with minimum and maximum MSE values, at the bottom of the table.

Given that the researcher does not know which simulated situation best represents their actual research situation, they first consider the estimator with the lowest overall average MSE. That is the AK1 estimator. It has an overall average value of 0.020. The next best estimator is the WAAP, with an overall average of 0.027. AK1 also takes on a relatively narrow range of values across the 30 experiments. Its minimum value is 0.001, and its maximum value is 0.081. This compares favorably with most of the other estimators, but not all. For example, Bom & Rachinger's EK estimator, while producing a slightly larger overall average value of MSE (0.028), takes on a narrower set of values (minimum = 0.008, maximum = 0.052). The WAAP and PP estimators have similar characteristics.

With respect to AK1, it is worth noting that simulations will tend to be biased toward selection models, because selection models have been designed to capture the very kinds of behaviors built into selection algorithms. This is not necessarily a bad thing. However, to the extent that actual publication selection behavior differs from simulated selection behavior, results may overstate the performance of selection models in real world datasets.

The researcher's choice comes down to a trade‐off between mean and dispersion, a choice that is complicated by the fact that randomness in the simulation process cautions against attaching too much significance to small numerical differences. We propose one possible solution, with the researcher choosing the AK1 estimator as best (yellow‐highlighted), while also choosing one or two other estimators (WAAP, PP, EK; highlighted in blue) for robustness checking.

## 7 CONCLUSION

The subject of MA estimator performance has received much attention in the literature (Alinaghi and Reed^2^; Bom and Rachinger^3^; Carter et al^4^; Hedges and Vevea^6^; McShane et al^7^; Moreno et al.,^8^; Rücker et al^10^; Simonsohn et al^11^; Stanley^12^; Stanley and Doucouliagos^13^; Stanley et al^1^; van Aert et al^14^; van Assen et al^15^). A goal of many of these studies has been to find a “best” estimator. However, there is an increasing awareness that no single estimator is “best” in all circumstances (Carter et al^4^). Unfortunately, the way previous studies have been conducted and reported has not been conducive to guiding meta‐analysts toward the best estimator for their particular research applications.

Different studies examine different sets of estimators, making it difficult to aggregate results across studies. They employ different experimental designs with different features, without being clear about which features are important for estimator performance. Finally, they typically do not make a distinction between the influence of observable and unobservable characteristics. For example, knowing that an estimator performs well when a MA sample is unaffected by publication selection, but poorly when it is affected, is not helpful if the meta‐analyst cannot observe whether their particular sample has this problem. What is missing is something akin to a flow‐chart that would map observable characteristics to experimental results which the meta‐analyst could then use to select the best estimator for their situation.

This study contributes toward that goal. We demonstrate how two characteristics that can be observed by the meta‐analyst – number of estimates in the meta‐analyst's sample (“sample size”) and the degree of effect heterogeneity, as measured by *I* ^*2*^ ‐ can be used to guide the meta‐analyst to experimental results that are most germane to their research application. We construct an extensive database of 1620 experiments and give an example how the database can be used to select a “best” estimator, or best set of estimators.

In our example of sample size = 100 and *I* ^*2*^ > 0.75. we find that Andrews and Kasy's symmetric selection estimator (“AK1”) performs best with respect to minimizing MSE, closely followed by Bom and Rachinger's “EK” estimator; Stanley, Doucouliagos, and Ioannidis' WAAP estimator; and Stanley and Doucouliagos' PET‐PEESE estimator. However, this example assumes the simulation design of Carter et al^4^. Other simulation designs with the same sample size and effect heterogeneity give different results.^9^ Thus, a major challenge going forward is to gain a better understanding of the factors that determine estimator performance.

A final contribution of our study is that we have made all of our experimental results publicly accessible via a ShinyApp at https://hong‐reed.shinyapps.io/HongReedInteractiveTables. Table 7 presented the results of 30 Monte Carlo experiments from the Carter, Schönbrodt, Gervais, and Hilgard^4^ simulation environment for sample sizes of 100 and high effect heterogeneity. The online results allow researchers to explore other scenarios that may be more relevant for their particular research situations.

## CONFLICT OF INTEREST

The authors declare no conflicts of interest.

## Supporting information


**Data S1.** Supplementary Information.

## Data Availability

All of the programming code and output files associated with this project are available at https://osf.io/pr4mb/.

## Endnotes

^1^ Hedges and Vevea^6^ estimate a 5PSM with the following four categories: (a) $\left({\hat{\beta }}_{i}/{\mathrm{SE}}_{i}\right)\geq 1.64$, (b) $\left({\hat{\beta }}_{i}/{\mathrm{SE}}_{i}\right)<0$, (c) $0\leq \left({\hat{\beta }}_{i}/{\mathrm{SE}}_{i}\right)<0.84,$ and (d) $0.84\leq \left({\hat{\beta }}_{i}/{\mathrm{SE}}_{i}\right)<1.64$.

^2^ For further details about the AK1 and AK2 estimators, see Hong et al.^21^

^3^ In a recent *Nature* study that compared meta‐analysis studies with related replications, Kvarven et al^23^ found that PET‐PEESE was better at reducing bias compared to several other popular estimators (Random Effects, Trim‐and‐Fill, 3PSM). However, it fared no better on the dimension of MSE.

^4^ We note that what SD&I call “50% Selection” does not imply that 50% of all estimates are filtered out of the meta‐analyst's sample. The percent of estimates actually impacted by selection bias depends on the size of the effect. For example, if the true effect is zero, what SD&I call “50% Selection” will result in 2.5% of all estimates being selected for the meta‐analyst's sample. At the other extreme, if the true effect is extremely large, “50% Selection” will result in 100% of all estimates being selected.

5 When there are more than one estimate per study, the selection rule needs to apply at the study level, as opposed to the level of individual estimates. In modeling the journal review process, we assume that journals typically do not say “we will publish your paper if you drop specific regressions.” Instead, they say “we will publish your paper, or not.” We chose 7 because, in previous research, it produced datasets that “looked like” real‐life meta‐analysis datasets (see Table 4 in Reed^9^).

6 Appendix 5 in Data S1 reports convergence rates for all the cells in Tables 3 and 4.

7 Note that the results for Type I Error Rate are restricted to those experiments for which the true effect = 0.

8 Results for |Coverage‐0.95| and Type I Error are reported in Appendix 3 in Data S1.

9 See Appendix 4 in Data S1 for a demonstration of how relative rankings of average performance vary across simulation environments even when the set of experiments are restricted to those having similar sample sizes and effect heterogeneity.
