# Supplementary material for: Using Monte Carlo experiments to select meta‐analytic estimators
Source: Res Synth Methods. 2020 Nov 17;12(2):192–215. doi: 10.1002/jrsm.1467 (PMC8074967; doi:10.1002/jrsm.1467)
Supplement: Supplementary file 1 — Data S1. Supplementary Information. [file JRSM-12-192-s001.docx]

**APPENDIX TO ‘USING MONTE CARLO EXPERIMENTS**

**TO SELECT META-ANALYTIC ESTIMATORS’**

by

Sanghyun Hong and W. Robert Reed

*Department of Economics and Finance*

*University of Canterbury*

*Christchurch, NEW ZEALAND*

**APPENDIX 1**

**Different Publication Selection Procedures in SD&I (2017)**

**
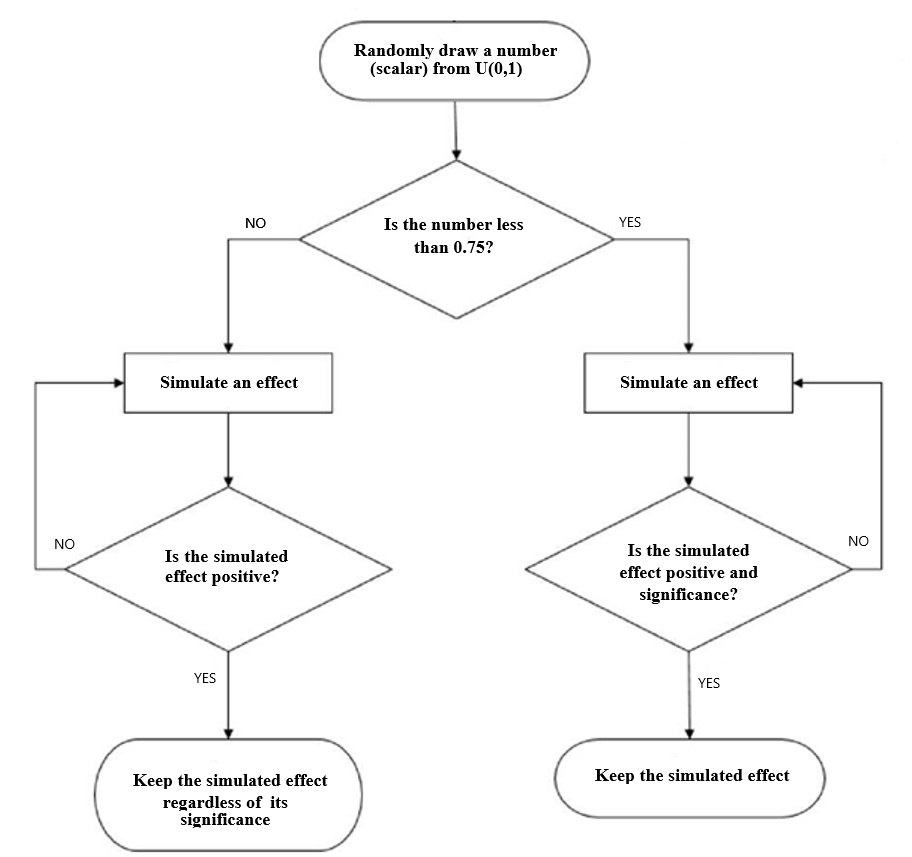
A. “50% Selective Reporting”**

**B. “75/100% Selective Reporting”**


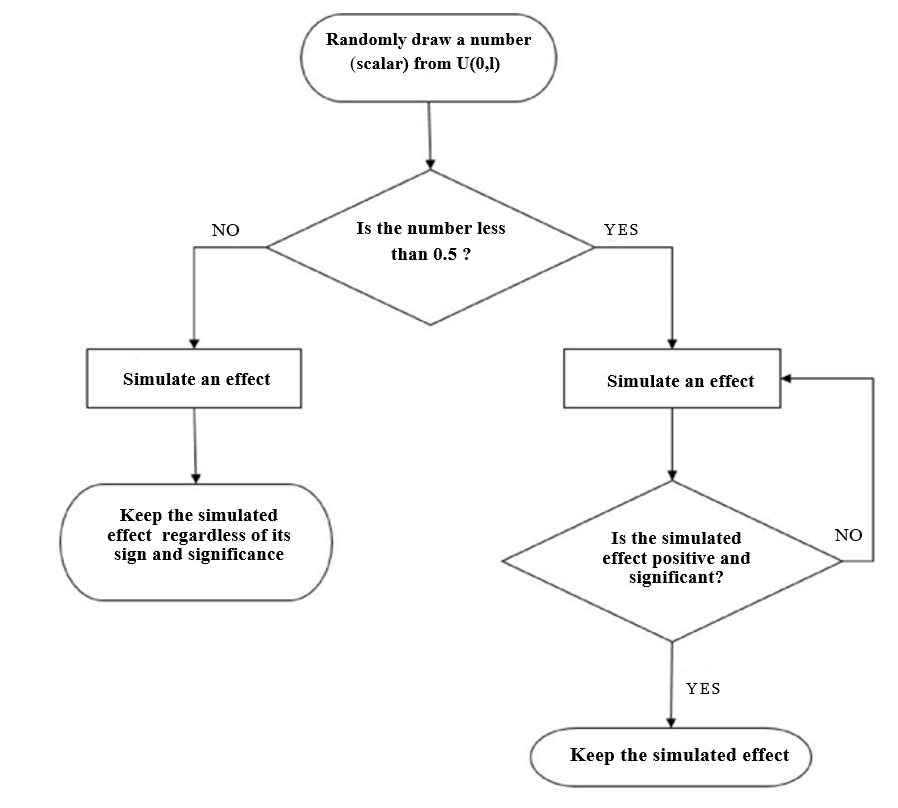


**APPENDIX 2**

**Description of Experiments**

| **STUDY: Stanley, Doucouliagos, & Ioannidis (2017). Finding the power to reduce publication bias. *Statistics in Medicine*.**  **TABLES: I, II, III, IV, V, VI** | | | | | | | |
| --- | --- | --- | --- | --- | --- | --- | --- |
|  | | ***Log Odds Ratio*** | | ***Cohen’s d*** | | | |
| ***Effect Size*** | | {0, 0.3, 0.54} | | {0, 0.5} | | | |
| ***Heterogeneity*** | | $\sigma_{h}=\left\{ 0, 0.006 \right\}$* | | {0, 6.25, 12.5, 25, 50} | | | |
| ***Publication Selection*** | | {0%, 50%}* | | {0%, 50%, 75/100%} | | | |
| ***Sample Size*** | | {5, 10, 20, 40, 80, 100, 200, 400, 800) | | {5, 10, 20, 40, 80, 100, 200, 400, 800) | | | |
| ***# of Experiments*** | | 3 x 2 x 9 = 54 | | 2 x 5 x 3 x 9 = 270 | | | |
| **STUDY: Alinaghi & Reed (2018). Meta-analysis and publication bias: How well does the FAT-PET-PEESE procedure work? *Research Synthesis Methods*.**  **TABLE: 6** | | | | | | | |
|  | | ***Regression (Random Effects)*** | | ***Regression (Panel Random Effects)*** | | | |
| ***Effect Size*** | | {0, 0.5, 1, 1.5, 2, 2.5, 3, 3.5, 4} | | {0, 0.5, 1, 1.5, 2, 2.5, 3, 3.5, 4} | | | |
| ***Heterogeneity*** | | Endogenous: *I^2^* > 75% | | Endogenous: *I^2^* > 75% | | | |
| ***Publication Selection*** | | {Significance, Correct Sign} | | {Significance, Correct Sign} | | | |
| ***Sample Size*** | | Endogenous: > 100 | | Endogenous: > 100 | | | |
| ***# of Experiments*** | | 9 x 2 = 18 | | 9 x 2 = 18 | | | |
| **STUDY: Bom and Rachinger (2019). A kinked meta‐regression model for publication bias correction. *Research Synthesis Methods.***  **TABLES: 1, 2, 3; FIGURES: 4, 5, 6, 7** | | | | | | | |
|  | ***Regression*** | | | |  | |  |
| ***Effect Size*** | {0, 1} | | | |  | |  |
| ***Heterogeneity*** | {0, 0.125, 0.25, 0.5, 1, 2, 4} | | | |  | |  |
| ***Publication Selection*** | {0%, 25%, 50%, 75%} | | | |  | |  |
| ***Sample Size*** | {5, 10, 20, 40, 80, 100, 200, 400, 800} | | | |  | |  |
| ***# of Experiments*** | 2 x 7 x 4 x 9 = 504 | | | |  | |  |
| **STUDY: Carter et al. (2019a). Correcting for Bias in Psychology: A Comparison of Meta-Analytic Methods. *Advances in Methods and Practices in Psychological Science.***  **TABLES: Reports stored online at https://osf.io/rf3ys** | | | | | | | |
|  | | | ***Cohen’s d*** | | |  |  |
| ***Effect Size*** | | | {0, 0.2, 0.5, 0.8} | | |  |  |
| ***Heterogeneity*** | | | {$\sigma_{h}=$0, 0.2, 0.4} | | |  |  |
| ***Publication Selection*** | | | {“No”, “Medium”, Strong”} | | |  |  |
| ***Questionable Research Practice*** | | | {“None”, “Medium”, High”} | | |  |  |
| ***Sample Size*** | | | {10, 30, 60, 100, 200, 400, 800) | | |  |  |
| ***# of Experiments*** | | | 4 x 3 x 3 x 3 x 7 = 756 | | |  |  |

*When publication selection is 0%, Stanley, Doucouliagos, and Ioannidis (2017) only allow $\sigma_{h}=0.006$; and when publication selection is 50%, they only allow $\sigma_{h}=0.006$.

**APPENDIX 3**

**Results on |Coverage-0.95| and Type I Error Rates to Complement TABLE 6**

**A. Sample Size = 10**

| **\|Coverage-0.95\|** | | | | | | ***Type I Error*** | | | | | |
| --- | --- | --- | --- | --- | --- | --- | --- | --- | --- | --- | --- |
| ***Low I^2^*** | | ***Moderate I^2^*** | | ***High I^2^*** | | ***Low I^2^*** | | ***Moderate I^2^*** | | ***High I^2^*** | |
| AK1 | 0.044 | pU | 0.114 | AK2 | 0.030 | pU | 0.011 | EK | 0.194 | AK2 | 0.026 |
| 3PSM | 0.053 | AK1 | 0.149 | AK1 | 0.114 | AK2 | 0.026 | pU | 0.200 | AK1 | 0.122 |
| pU | 0.060 | EK | 0.186 | WAAP | 0.120 | EK | 0.059 | PP | 0.227 | WAAP | 0.203 |
| WAAP | 0.076 | 3PSM | 0.186 | TF | 0.130 | PP | 0.062 | 3PSM | 0.260 | EK | 0.205 |
| TF | 0.102 | WAAP | 0.207 | EK | 0.159 | AK1 | 0.092 | AK2 | 0.263 | TF | 0.221 |
| PP | 0.110 | PP | 0.236 | PP | 0.184 | 3PSM | 0.114 | AK1 | 0.298 | PP | 0.237 |
| AK2 | 0.127 | TF | 0.289 | RE | 0.223 | 4PSM | 0.181 | 4PSM | 0.378 | 3PSM | 0.266 |
| EK | 0.153 | AK2 | 0.320 | pU | 0.225 | WAAP | 0.267 | WAAP | 0.484 | 4PSM | 0.297 |
| 4PSM | 0.162 | 4PSM | 0.344 | 3PSM | 0.232 | TF | 0.273 | TF | 0.549 | pU | 0.323 |
| RE | 0.207 | RE | 0.402 | 4PSM | 0.237 | RE | 0.322 | RE | 0.705 | RE | 0.358 |
| pC | NA | pC | NA | pC | NA | pC | NA | pC | NA | pC | NA |

**B. Sample Size = 30**

| **\|Coverage-0.95\|** | | | | | | ***Type I Error*** | | | | | |
| --- | --- | --- | --- | --- | --- | --- | --- | --- | --- | --- | --- |
| ***Low I^2^*** | | ***Moderate I^2^*** | | ***High I^2^*** | | ***Low I^2^*** | | ***Moderate I^2^*** | | ***High I^2^*** | |
| WAAP | 0.049 | 3PSM | 0.143 | AK2 | 0.060 | pU | 0.016 | pU | 0.146 | AK2 | 0.035 |
| 3PSM | 0.052 | pU | 0.154 | 4PSM | 0.182 | AK2 | 0.036 | AK2 | 0.201 | 3PSM | 0.265 |
| AK1 | 0.052 | AK2 | 0.238 | 3PSM | 0.187 | EK | 0.052 | 3PSM | 0.227 | 4PSM | 0.272 |
| 4PSM | 0.086 | AK1 | 0.274 | WAAP | 0.234 | PP | 0.057 | EK | 0.263 | EK | 0.353 |
| TF | 0.095 | WAAP | 0.274 | EK | 0.301 | 4PSM | 0.068 | PP | 0.288 | WAAP | 0.365 |
| PP | 0.104 | EK | 0.317 | AK1 | 0.348 | TF | 0.145 | 4PSM | 0.385 | PP | 0.386 |
| AK2 | 0.112 | PP | 0.333 | TF | 0.351 | 3PSM | 0.152 | WAAP | 0.562 | TF | 0.532 |
| pU | 0.204 | 4PSM | 0.345 | PP | 0.363 | AK1 | 0.174 | TF | 0.746 | pU | 0.582 |
| RE | 0.268 | TF | 0.397 | pU | 0.401 | WAAP | 0.227 | AK1 | 0.766 | AK1 | 0.617 |
| EK | 0.284 | RE | 0.590 | RE | 0.433 | RE | 0.279 | RE | 0.794 | RE | 0.658 |
| pC | NA | pC | NA | pC | NA | pC | NA | pC | NA | pC | NA |

**C. Sample Size = 60**

| **\|Coverage-0.95\|** | | | | | | ***Type I Error*** | | | | | |
| --- | --- | --- | --- | --- | --- | --- | --- | --- | --- | --- | --- |
| ***Low I^2^*** | | ***Moderate I^2^*** | | ***High I^2^*** | | ***Low I^2^*** | | ***Moderate I^2^*** | | ***High I^2^*** | |
| WAAP | 0.058 | 3PSM | 0.168 | AK2 | 0.124 | pU | 0.013 | pU | 0.185 | AK2 | 0.080 |
| AK1 | 0.078 | AK2 | 0.220 | 4PSM | 0.252 | AK2 | 0.077 | 3PSM | 0.270 | 4PSM | 0.347 |
| 3PSM | 0.085 | pU | 0.268 | WAAP | 0.279 | EK | 0.078 | AK2 | 0.280 | 3PSM | 0.360 |
| 4PSM | 0.094 | WAAP | 0.305 | 3PSM | 0.284 | PP | 0.081 | EK | 0.331 | EK | 0.412 |
| AK2 | 0.098 | AK1 | 0.320 | EK | 0.364 | 4PSM | 0.085 | PP | 0.349 | WAAP | 0.414 |
| TF | 0.110 | 4PSM | 0.381 | PP | 0.421 | TF | 0.197 | 4PSM | 0.432 | PP | 0.446 |
| PP | 0.148 | PP | 0.388 | AK1 | 0.423 | 3PSM | 0.239 | WAAP | 0.528 | TF | 0.647 |
| RE | 0.356 | EK | 0.397 | TF | 0.448 | AK1 | 0.254 | AK1 | 0.776 | AK1 | 0.705 |
| pU | 0.393 | TF | 0.445 | RE | 0.521 | WAAP | 0.279 | TF | 0.778 | RE | 0.715 |
| EK | 0.410 | RE | 0.666 | pU | 0.523 | RE | 0.322 | RE | 0.790 | pU | 0.764 |
| pC | NA | pC | NA | pC | NA | pC | NA | pC | NA | pC | NA |

**D. Sample Size = 100**

| **\|Coverage-0.95\|** | | | | | | ***Type I Error*** | | | | | |
| --- | --- | --- | --- | --- | --- | --- | --- | --- | --- | --- | --- |
| ***Low I^2^*** | | ***Moderate I^2^*** | | ***High I^2^*** | | ***Low I^2^*** | | ***Moderate I^2^*** | | ***High I^2^*** | |
| WAAP | 0.064 | 3PSM | 0.218 | AK2 | 0.173 | pU | 0.009 | pU | 0.132 | AK2 | 0.137 |
| AK1 | 0.107 | AK2 | 0.294 | WAAP | 0.349 | EK | 0.105 | 3PSM | 0.313 | 3PSM | 0.441 |
| 3PSM | 0.130 | WAAP | 0.326 | 4PSM | 0.364 | PP | 0.110 | AK2 | 0.318 | EK | 0.448 |
| 4PSM | 0.134 | AK1 | 0.345 | 3PSM | 0.381 | AK2 | 0.115 | EK | 0.378 | 4PSM | 0.451 |
| TF | 0.143 | pU | 0.362 | EK | 0.404 | 4PSM | 0.123 | PP | 0.385 | WAAP | 0.469 |
| AK2 | 0.152 | PP | 0.432 | PP | 0.468 | TF | 0.254 | WAAP | 0.519 | PP | 0.480 |
| PP | 0.217 | TF | 0.441 | AK1 | 0.534 | AK1 | 0.290 | 4PSM | 0.527 | TF | 0.776 |
| RE | 0.446 | 4PSM | 0.442 | TF | 0.592 | WAAP | 0.306 | TF | 0.713 | pU | 0.776 |
| EK | 0.526 | EK | 0.449 | RE | 0.598 | 3PSM | 0.340 | AK1 | 0.714 | AK1 | 0.776 |
| pU | 0.532 | RE | 0.703 | pU | 0.614 | RE | 0.363 | RE | 0.738 | RE | 0.785 |
| pC | NA | pC | NA | pC | NA | pC | NA | pC | NA | pC | NA |

**E. Sample Size = 200**

| **\|Coverage-0.95\|** | | | | | | ***Type I Error*** | | | | | |
| --- | --- | --- | --- | --- | --- | --- | --- | --- | --- | --- | --- |
| ***Low I^2^*** | | ***Moderate I^2^*** | | ***High I^2^*** | | ***Low I^2^*** | | ***Moderate I^2^*** | | ***High I^2^*** | |
| WAAP | 0.078 | 3PSM | 0.347 | AK2 | 0.339 | pU | 0.007 | pU | 0.186 | AK2 | 0.255 |
| TF | 0.160 | WAAP | 0.389 | WAAP | 0.401 | EK | 0.179 | 3PSM | 0.354 | WAAP | 0.497 |
| AK1 | 0.188 | AK1 | 0.445 | EK | 0.461 | PP | 0.181 | AK2 | 0.389 | EK | 0.512 |
| AK2 | 0.197 | AK2 | 0.492 | 3PSM | 0.499 | AK2 | 0.207 | EK | 0.500 | PP | 0.540 |
| 4PSM | 0.220 | PP | 0.498 | 4PSM | 0.534 | 4PSM | 0.222 | PP | 0.507 | 3PSM | 0.585 |
| 3PSM | 0.222 | pU | 0.501 | PP | 0.534 | TF | 0.332 | 4PSM | 0.569 | 4PSM | 0.644 |
| PP | 0.366 | 4PSM | 0.520 | AK1 | 0.623 | AK1 | 0.347 | WAAP | 0.589 | AK1 | 0.783 |
| RE | 0.550 | TF | 0.531 | RE | 0.634 | WAAP | 0.359 | TF | 0.716 | RE | 0.798 |
| pU | 0.643 | EK | 0.537 | TF | 0.694 | RE | 0.451 | AK1 | 0.727 | TF | 0.815 |
| EK | 0.653 | RE | 0.782 | pU | 0.708 | 3PSM | 0.477 | RE | 0.766 | pU | 0.847 |
| pC | NA | pC | NA | pC | NA | pC | NA | pC | NA | pC | NA |

**F. Sample Size = 400**

| **\|Coverage-0.95\|** | | | | | | ***Type I Error*** | | | | | |
| --- | --- | --- | --- | --- | --- | --- | --- | --- | --- | --- | --- |
| ***Low I^2^*** | | ***Moderate I^2^*** | | ***High I^2^*** | | ***Moderate I^2^*** | | ***Low I^2^*** | | ***High I^2^*** | |
| WAAP | 0.108 | WAAP | 0.459 | WAAP | 0.472 | pU | 0.005 | pU | 0.275 | AK2 | 0.443 |
| TF | 0.223 | 3PSM | 0.523 | AK2 | 0.496 | EK | 0.284 | 3PSM | 0.428 | WAAP | 0.540 |
| AK1 | 0.308 | AK2 | 0.549 | EK | 0.541 | PP | 0.288 | AK2 | 0.440 | EK | 0.594 |
| 4PSM | 0.339 | AK1 | 0.566 | 3PSM | 0.586 | AK2 | 0.335 | 4PSM | 0.593 | PP | 0.616 |
| AK2 | 0.353 | PP | 0.584 | PP | 0.617 | 4PSM | 0.354 | EK | 0.645 | 3PSM | 0.714 |
| 3PSM | 0.358 | 4PSM | 0.608 | 4PSM | 0.630 | TF | 0.407 | PP | 0.651 | 4PSM | 0.789 |
| PP | 0.550 | pU | 0.611 | AK1 | 0.669 | WAAP | 0.445 | WAAP | 0.683 | AK1 | 0.796 |
| RE | 0.666 | TF | 0.633 | RE | 0.712 | AK1 | 0.460 | TF | 0.721 | RE | 0.820 |
| pU | 0.712 | EK | 0.639 | pU | 0.766 | RE | 0.568 | AK1 | 0.756 | TF | 0.860 |
| EK | 0.730 | RE | 0.833 | TF | 0.777 | 3PSM | 0.602 | RE | 0.815 | pU | 0.877 |
| pC | NA | pC | NA | pC | NA | pC | NA | pC | NA | pC | NA |

**G. Sample Size = 800**

| **\|Coverage-0.95\|** | | | | | | ***Type I Error*** | | | | | |
| --- | --- | --- | --- | --- | --- | --- | --- | --- | --- | --- | --- |
| ***Low I^2^*** | | ***Moderate I^2^*** | | ***High I^2^*** | | ***Low I^2^*** | | ***Moderate I^2^*** | | ***High I^2^*** | |
| WAAP | 0.148 | WAAP | 0.532 | WAAP | 0.549 | pU | 0.001 | pU | 0.359 | WAAP | 0.605 |
| TF | 0.316 | AK2 | 0.605 | AK2 | 0.605 | EK | 0.384 | AK2 | 0.499 | AK2 | 0.648 |
| 4PSM | 0.466 | 3PSM | 0.652 | EK | 0.624 | PP | 0.385 | 3PSM | 0.538 | EK | 0.667 |
| AK1 | 0.466 | AK1 | 0.653 | 3PSM | 0.642 | AK2 | 0.433 | 4PSM | 0.628 | PP | 0.685 |
| AK2 | 0.471 | 4PSM | 0.666 | 4PSM | 0.684 | 4PSM | 0.479 | TF | 0.727 | 3PSM | 0.788 |
| 3PSM | 0.495 | PP | 0.672 | PP | 0.692 | WAAP | 0.514 | EK | 0.731 | AK1 | 0.822 |
| PP | 0.689 | pU | 0.706 | AK1 | 0.708 | TF | 0.528 | PP | 0.736 | 4PSM | 0.832 |
| pU | 0.756 | EK | 0.730 | RE | 0.773 | AK1 | 0.602 | WAAP | 0.756 | RE | 0.858 |
| EK | 0.761 | TF | 0.745 | pU | 0.839 | 3PSM | 0.659 | AK1 | 0.805 | pU | 0.897 |
| RE | 0.764 | RE | 0.862 | TF | 0.844 | RE | 0.666 | RE | 0.863 | TF | 0.923 |
| pC | NA | pC | NA | pC | NA | pC | NA | pC | NA | pC | NA |

NOTE: The panels above rank the performance of the eleven estimators on the basis of their average |Coverage-0.95| and Type I Error rate performance, disaggregated by {sample size, effect heterogeneity} categories. Estimators are ranked from “best” (smallest deviation between coverage rates and 95%, Type I error rate closest to 5%) to worst. Values in the tables are the average values for the respective performance measures and {sample size, effect heterogeneity} categories. For both |Coverage-0.95| and Type I Error rate, the top two estimators in the cell for smallest sample size (10) and effect heterogeneity (low *I^2^*) are identified by color-coding. For |Coverage-0.95|, these are the AK1 and 3PSM estimators. For Type I Error rate, they are pU and AK2. The relative position of these estimators are then tracked as sample size and effect heterogeneity increases.

**APPENDIX 4**

**Comparison of Estimator Performance across Simulation Environments:**

**Sample Size ≥ 100 and *I^2^* > 0.75**

**A. |Bias|**

| ***SD&I (2017)*** | | ***A&R (2018)*** | | | ***B&R (2019)*** | | | ***CSG&H (2019a)*** | |
| --- | --- | --- | --- | --- | --- | --- | --- | --- | --- |
| AK2 | 0.012 | | EK | 0.202 | | EK | 0.08 | EK | 0.071 |
| 4PSM | 0.024 | | AK2 | 0.206 | | AK2 | 0.098 | PP | 0.09 |
| 3PSM | 0.047 | | PP | 0.245 | | 4PSM | 0.18 | WAAP | 0.099 |
| EK | 0.079 | | WAAP | 0.252 | | PP | 0.185 | AK1 | 0.108 |
| PP | 0.091 | | TF | 0.274 | | 3PSM | 0.262 | 3PSM | 0.136 |
| AK1 | 0.098 | | 4PSM | 0.306 | | TF | 0.29 | TF | 0.15 |
| WAAP | 0.112 | | AK1 | 0.371 | | WAAP | 0.294 | AK2 | 0.153 |
| TF | 0.116 | | 3PSM | 0.451 | | pU | 0.385 | 4PSM | 0.188 |
| RE | 0.144 | | RE | 0.54 | | pC | 0.389 | pU | 0.191 |
| pU | 0.254 | | pC | 1.562 | | AK1 | 0.417 | pC | 0.203 |
| pC | 0.257 | | pU | 1.59 | | RE | 0.548 | RE | 0.22 |

**B. MSE**

| ***SD&I (2017)*** | | ***A&R (2018)*** | | | ***B&R (2019)*** | | | ***CSG&H (2019a)*** | |
| --- | --- | --- | --- | --- | --- | --- | --- | --- | --- |
| AK2 | 0.003 | | AK2 | 0.235 | | EK | 0.034 | EK | 0.017 |
| 4PSM | 0.006 | | TF | 0.24 | | PP | 0.106 | AK1 | 0.018 |
| 3PSM | 0.015 | | 4PSM | 0.327 | | AK2 | 0.11 | PP | 0.021 |
| EK | 0.019 | | AK1 | 0.343 | | 4PSM | 0.153 | WAAP | 0.022 |
| PP | 0.026 | | WAAP | 0.422 | | pU | 0.175 | 3PSM | 0.033 |
| AK1 | 0.029 | | 3PSM | 0.448 | | pC | 0.185 | TF | 0.034 |
| WAAP | 0.032 | | PP | 0.456 | | WAAP | 0.196 | AK2 | 0.039 |
| TF | 0.034 | | RE | 0.474 | | 3PSM | 0.23 | pU | 0.054 |
| RE | 0.048 | | EK | 0.572 | | TF | 0.242 | 4PSM | 0.055 |
| pU | 0.094 | | pC | 3.613 | | AK1 | 0.458 | pC | 0.059 |
| pC | 0.096 | | pU | 3.726 | | RE | 0.726 | RE | 0.079 |

**C. |Coverage-0.95|***

| ***SD&I (2017)*** | | ***A&R (2018)*** | | | ***B&R (2019)*** | | | ***CSG&H (2019a)*** | |
| --- | --- | --- | --- | --- | --- | --- | --- | --- | --- |
| AK2 | 0.032 | | AK2 | 0.275 | | AK2 | 0.037 | AK2 | 0.407 |
| 3PSM | 0.146 | | 4PSM | 0.377 | | 4PSM | 0.073 | WAAP | 0.446 |
| 4PSM | 0.193 | | AK1 | 0.531 | | EK | 0.15 | EK | 0.511 |
| EK | 0.303 | | TF | 0.546 | | 3PSM | 0.155 | 3PSM | 0.531 |
| PP | 0.363 | | WAAP | 0.582 | | PP | 0.317 | 4PSM | 0.558 |
| AK1 | 0.416 | | 3PSM | 0.59 | | TF | 0.523 | PP | 0.582 |
| WAAP | 0.501 | | EK | 0.695 | | WAAP | 0.57 | AK1 | 0.636 |
| TF | 0.559 | | PP | 0.705 | | AK1 | 0.578 | RE | 0.682 |
| RE | 0.601 | | RE | 0.814 | | RE | 0.676 | TF | 0.731 |
| pU | 0.902 | | pU | 0.923 | | pU | 0.95 | pU | 0.735 |
| pC | NA | | pC | NA | | pC | NA | pC | NA |

**D. Type 1 Error****

| ***SD&I (2017)*** | | ***A&R (2018)*** | | | ***B&R (2019)*** | | | ***CSG&H (2019a)*** | |
| --- | --- | --- | --- | --- | --- | --- | --- | --- | --- |
| AK2 | 0.026 | | AK2 | 0.391 | | AK2 | 0.027 | AK2 | 0.360 |
| 4PSM | 0.204 | | AK1 | 0.505 | | 4PSM | 0.089 | WAAP | 0.528 |
| 3PSM | 0.212 | | RE | 0.507 | | 3PSM | 0.183 | EK | 0.555 |
| EK | 0.474 | | TF | 0.516 | | EK | 0.256 | PP | 0.580 |
| PP | 0.513 | | WAAP | 0.643 | | PP | 0.325 | 3PSM | 0.632 |
| RE | 0.602 | | PP | 0.722 | | TF | 0.653 | 4PSM | 0.679 |
| AK1 | 0.602 | | EK | 0.729 | | WAAP | 0.689 | AK1 | 0.794 |
| WAAP | 0.616 | | 4PSM | 0.791 | | AK1 | 0.764 | RE | 0.815 |
| TF | 0.733 | | 3PSM | 0.892 | | RE | 0.765 | TF | 0.843 |
| pU | 1.000 | | pU | 1.000 | | pU | 1.000 | pU | 0.849 |
| pC | NA | | pC | NA | | pC | NA | pC | NA |

NOTE: The four panels rank the performance of the eleven estimators on the basis of their average Bias, MSE, |Coverage-0.95|, and Type I Error performance, disaggregated by simulation environment. Estimators are ranked from “best” (least Bias, smallest MSE, etc.) to worst. Values in the tables are the average values for the respective performance measures and simulation environments. In each panel the best and second best performing estimators in the SD&I environments are color-coded brown and grey, respectively. This allows one to track their relative performance across the remaining three simulation environments. The difference between this table and TABLE 4 is that this table only reports results for those experiments where Sample Size ≥ 100 and *I^2^* > 0.75

* This column reports the average, absolute value of the difference between (i) the percent of times the 95% confidence interval contains the true mean value and (ii) 95%.

** This column reports the percentage of false positives when the true mean effect = 0; i.e., the percent of times an estimate is statistically significant when there is no true effect

**APPENDIX 5**

**A. Convergence Rates for Bias/MSE/Coverage Rate Calculations: All Experiments and by Simulation Environment**

| **All** | | ***SD&I (2017)*** | | ***A&R (2018)*** | | ***B&R (2019)*** | | ***CSG&H (2019a)*** | |
| --- | --- | --- | --- | --- | --- | --- | --- | --- | --- |
| 3PSM | 0.9244 | 3PSM | 0.9925 | 3PSM | 1.0000 | 3PSM | 0.9968 | 3PSM | 0.8434 |
| 4PSM | 0.9288 | 4PSM | 0.9997 | 4PSM | 1.0000 | 4PSM | 0.9999 | 4PSM | 0.8476 |
| AK1 | 0.9976 | AK1 | 0.9937 | AK1 | 1.0000 | AK1 | 0.9980 | AK1 | 0.9990 |
| AK2 | 0.3760 | AK2 | 0.3527 | AK2 | 0.7298 | AK2 | 0.5374 | AK2 | 0.2616 |
| EK | 1.0000 | EK | 1.0000 | EK | 1.0000 | EK | 1.0000 | EK | 1.0000 |
| pC | 0.9871 | pC | 0.9676 | pC | 1.0000 | pC | 0.9874 | pC | 0.9947 |
| pU | 0.9871 | pU | 0.9676 | pU | 1.0000 | pU | 0.9874 | pU | 0.9947 |
| PP | 1.0000 | PP | 1.0000 | PP | 1.0000 | PP | 1.0000 | PP | 1.0000 |
| RE | 1.0000 | RE | 1.0000 | RE | 1.0000 | RE | 1.0000 | RE | 1.0000 |
| TF | 0.9798 | TF | 0.9998 | TF | 1.0000 | TF | 0.9998 | TF | 0.9568 |
| WAAP | 1.0000 | WAAP | 1.0000 | WAAP | 1.0000 | WAAP | 1.0000 | WAAP | 1.0000 |

.

NOTE: Red color-coding indicates a convergence rate less than 90%. Yellow color-coding indicates a convergence rate of 90-98%.

**B. Convergence Rates for Type I Error Rate calculations: All Experiments and by Simulation Environment**

| **All** | | ***SD&I (2017)*** | | ***A&R (2018)*** | | ***B&R (2019)*** | | ***CSG&H (2019a)*** | |
| --- | --- | --- | --- | --- | --- | --- | --- | --- | --- |
| 3PSM | 0.944 | 3PSM | 0.985 | 3PSM | 1.000 | 3PSM | 0.994 | 3PSM | 0.843 |
| 4PSM | 0.952 | 4PSM | 1.000 | 4PSM | 1.000 | 4PSM | 1.000 | 4PSM | 0.848 |
| AK1 | 0.994 | AK1 | 0.989 | AK1 | 1.000 | AK1 | 0.996 | AK1 | 0.997 |
| AK2 | 0.569 | AK2 | 0.490 | AK2 | 0.987 | AK2 | 0.697 | AK2 | 0.453 |
| EK | 1.000 | EK | 1.000 | EK | 1.000 | EK | 1.000 | EK | 1.000 |
| pC | 0.967 | pC | 0.936 | pC | 1.000 | pC | 0.975 | pC | 0.981 |
| pU | 0.967 | pU | 0.936 | pU | 1.000 | pU | 0.975 | pU | 0.981 |
| PP | 1.000 | PP | 1.000 | PP | 1.000 | PP | 1.000 | PP | 1.000 |
| RE | 1.000 | RE | 1.000 | RE | 1.000 | RE | 1.000 | RE | 1.000 |
| TF | 0.993 | TF | 1.000 | TF | 1.000 | TF | 1.000 | TF | 0.979 |
| WAAP | 1.000 | WAAP | 1.000 | WAAP | 1.000 | WAAP | 1.000 | WAAP | 1.000 |

.

NOTE: Red color-coding indicates a convergence rate less than 90%. Yellow color-coding indicates a convergence rate of 90-98%.

**C. Convergence Rates for CSG&H (2019a) Simulation Environment by**

**Sample Size and *I^2^***

| ***Low I^2^*** | | ***Moderate I^2^*** | | ***High I^2^*** | | ***Low I^2^*** | | ***Moderate I^2^*** | | ***High I^2^*** | |
| --- | --- | --- | --- | --- | --- | --- | --- | --- | --- | --- | --- |
| **Sample Size = 10** | | | | | | **Sample Size = 30** | | | | | |
| 3PSM | 0.9188 | 3PSM | 0.9736 | 3PSM | 0.9929 | 3PSM | 0.8291 | 3PSM | 0.9681 | 3PSM | 0.9993 |
| 4PSM | 0.9295 | 4PSM | 0.9772 | 4PSM | 0.9953 | 4PSM | 0.8328 | 4PSM | 0.9687 | 4PSM | 0.9994 |
| AK1 | 0.9867 | AK1 | 0.9971 | AK1 | 0.9997 | AK1 | 0.9972 | AK1 | 1.0000 | AK1 | 0.9999 |
| AK2 | 0.0577 | AK2 | 0.0763 | AK2 | 0.3572 | AK2 | 0.1056 | AK2 | 0.1127 | AK2 | 0.4722 |
| EK | 1.0000 | EK | 1.0000 | EK | 1.0000 | EK | 1.0000 | EK | 1.0000 | EK | 1.0000 |
| pC | 0.9490 | pC | 0.9814 | pC | 0.9872 | pC | 0.9792 | pC | 0.9983 | pC | 0.9997 |
| pU | 0.9490 | pU | 0.9814 | pU | 0.9872 | pU | 0.9792 | pU | 0.9983 | pU | 0.9997 |
| PP | 1.0000 | PP | 1.0000 | PP | 1.0000 | PP | 1.0000 | PP | 1.0000 | PP | 1.0000 |
| RE | 1.0000 | RE | 1.0000 | RE | 1.0000 | RE | 1.0000 | RE | 1.0000 | RE | 1.0000 |
| TF | 0.9701 | TF | 0.9735 | TF | 0.9979 | TF | 0.9399 | TF | 0.9229 | TF | 0.9941 |
| WAAP | 1.0000 | WAAP | 1.0000 | WAAP | 1.0000 | WAAP | 1.0000 | WAAP | 1.0000 | WAAP | 1.0000 |
| **Sample Size = 60** | | | | | | **Sample Size = 100** | | | | | |
| 3PSM | 0.7143 | 3PSM | 0.9522 | 3PSM | 0.9999 | 3PSM | 0.5979 | 3PSM | 0.9304 | 3PSM | 1.0000 |
| 4PSM | 0.7164 | 4PSM | 0.9527 | 4PSM | 1.0000 | 4PSM | 0.6035 | 4PSM | 0.9310 | 4PSM | 1.0000 |
| AK1 | 0.9995 | AK1 | 1.0000 | AK1 | 0.9999 | AK1 | 0.9999 | AK1 | 1.0000 | AK1 | 0.9999 |
| AK2 | 0.1364 | AK2 | 0.1398 | AK2 | 0.5350 | AK2 | 0.1649 | AK2 | 0.1715 | AK2 | 0.5344 |
| EK | 1.0000 | EK | 1.0000 | EK | 1.0000 | EK | 1.0000 | EK | 1.0000 | EK | 1.0000 |
| pC | 0.9906 | pC | 0.9998 | pC | 1.0000 | pC | 0.9961 | pC | 1.0000 | pC | 1.0000 |
| pU | 0.9906 | pU | 0.9998 | pU | 1.0000 | pU | 0.9961 | pU | 1.0000 | pU | 1.0000 |
| PP | 1.0000 | PP | 1.0000 | PP | 1.0000 | PP | 1.0000 | PP | 1.0000 | PP | 1.0000 |
| RE | 1.0000 | RE | 1.0000 | RE | 1.0000 | RE | 1.0000 | RE | 1.0000 | RE | 1.0000 |
| TF | 0.9353 | TF | 0.9114 | TF | 0.9984 | TF | 0.9366 | TF | 0.9125 | TF | 0.9996 |
| WAAP | 1.0000 | WAAP | 1.0000 | WAAP | 1.0000 | WAAP | 1.0000 | WAAP | 1.0000 | WAAP | 1.0000 |
| **Sample Size = 200** | | | | | | **Sample Size = 400** | | | | | |
| 3PSM | 0.4112 | 3PSM | 0.8861 | 3PSM | 1.0000 | 3PSM | 0.2334 | 3PSM | 0.8440 | 3PSM | 1.0000 |
| 4PSM | 0.4223 | 4PSM | 0.8897 | 4PSM | 1.0000 | 4PSM | 0.2519 | 4PSM | 0.8497 | 4PSM | 1.0000 |
| AK1 | 0.9999 | AK1 | 1.0000 | AK1 | 0.9998 | AK1 | 0.9999 | AK1 | 1.0000 | AK1 | 0.9994 |
| AK2 | 0.2046 | AK2 | 0.2091 | AK2 | 0.5915 | AK2 | 0.2440 | AK2 | 0.2393 | AK2 | 0.6060 |
| EK | 1.0000 | EK | 1.0000 | EK | 1.0000 | EK | 1.0000 | EK | 1.0000 | EK | 1.0000 |
| pC | 0.9997 | pC | 1.0000 | pC | 1.0000 | pC | 1.0000 | pC | 1.0000 | pC | 1.0000 |
| pU | 0.9997 | pU | 1.0000 | pU | 1.0000 | pU | 1.0000 | pU | 1.0000 | pU | 1.0000 |
| PP | 1.0000 | PP | 1.0000 | PP | 1.0000 | PP | 1.0000 | PP | 1.0000 | PP | 1.0000 |
| RE | 1.0000 | RE | 1.0000 | RE | 1.0000 | RE | 1.0000 | RE | 1.0000 | RE | 1.0000 |
| TF | 0.9458 | TF | 0.9314 | TF | 1.0000 | TF | 0.9615 | TF | 0.9482 | TF | 1.0000 |
| WAAP | 1.0000 | WAAP | 1.0000 | WAAP | 1.0000 | WAAP | 1.0000 | WAAP | 1.0000 | WAAP | 1.0000 |

| ***Low I^2^*** | | ***Moderate I^2^*** | | ***High I^2^*** | |
| --- | --- | --- | --- | --- | --- |
| **Sample Size = 800** | | | | | |
| 3PSM | 0.1266 | 3PSM | 0.8250 | 3PSM | 1.0000 |
| 4PSM | 0.1469 | 4PSM | 0.8321 | 4PSM | 1.0000 |
| AK1 | 0.9998 | AK1 | 1.0000 | AK1 | 0.9989 |
| AK2 | 0.2840 | AK2 | 0.2733 | AK2 | 0.6449 |
| EK | 1.0000 | EK | 1.0000 | EK | 1.0000 |
| pC | 1.0000 | pC | 1.0000 | pC | 1.0000 |
| pU | 1.0000 | pU | 1.0000 | pU | 1.0000 |
| PP | 1.0000 | PP | 1.0000 | PP | 1.0000 |
| RE | 1.0000 | RE | 1.0000 | RE | 1.0000 |
| TF | 0.9804 | TF | 0.9648 | TF | 1.0000 |
| WAAP | 1.0000 | WAAP | 1.0000 | WAAP | 1.0000 |

NOTE: Red color-coding indicates a convergence rate less than 90%. Yellow color-coding indicates a convergence rate of 90-98%.
